# Continuous norming of psychometric tests: A simulation study of parametric and semi-parametric approaches

**DOI:** 10.1371/journal.pone.0222279

**Published:** 2019-09-17

**Authors:** Alexandra Lenhard, Wolfgang Lenhard, Sebastian Gary

**Affiliations:** 1 Test Development Center, Psychometrica, Dettelbach, Bavaria, Germany; 2 Institute of Psychology, University of Wuerzburg, Bavaria, Germany; University of Copenhagen, DENMARK

## Abstract

Continuous norming methods have seldom been subjected to scientific review. In this simulation study, we compared parametric with semi-parametric continuous norming methods in psychometric tests by constructing a fictitious population model within which a latent ability increases with age across seven age groups. We drew samples of different sizes (*n* = 50, 75, 100, 150, 250, 500 and 1,000 per age group) and simulated the results of an easy, medium, and difficult test scale based on Item Response Theory (IRT). We subjected the resulting data to different continuous norming methods and compared the data fit under the different test conditions with a representative cross-validation dataset of *n* = 10,000 per age group. The most significant differences were found in suboptimal (i.e., too easy or too difficult) test scales and in ability levels that were far from the population mean. We discuss the results with regard to the selection of the appropriate modeling techniques in psychometric test construction, the required sample sizes, and the requirement to report appropriate quantitative and qualitative test quality criteria for continuous norming methods in test manuals.

## Introduction

Precise and reliable norm scores are an important quality criterion for psychometric tests, because critical life decisions, such as school placement decisions, rehabilitative treatment, and psychotherapeutic intervention decisions, are often based on the results of psychometric tests. In many countries, supportive measures for children with intellectual disabilities are almost exclusively restricted to children with a general IQ below 70. The granting of support measures, therapy, or compensation for specific learning disorders at school also regularly depends on whether a normed score of an appropriate test of reading, writing, or mathematics falls below a certain threshold [1]. Furthermore, strict cutoffs play a vital role in epidemiologic studies (e.g., in the seminal Isle of Wight studies [2]). Even in court decisions, an IQ score of 70 is used as a threshold to assess the accountability of a defendant. Given the importance of such decisions, a psychometric test should not only possess sufficient reliability and validity of the obtained raw scores, but it should also be able to precisely rank the raw scores in relation to the reference sample.

### Conventional and continuous norming

Whenever there are precise criteria on how raw scores can directly be interpreted, as is the case with criterion or mastery-referenced tests, there is no need to convert these raw scores into norm scores. For example, a criterion-referenced reading test could require a child to read aloud a text within a certain time and without exceeding a defined number of errors. However, the vast majority of psychometric tests aim to classify a test result in relation to a reference population. Within this scope, raw scores of a certain test can only be interpreted meaningfully, if population-based comparative scores–i.e., norm scores—are available [3]. Norm Scores indicate how the raw scores of a test are distributed in the population, that is, they are an estimator for the probability of a specific test result in the population. To produce the norm tables, the empirical cumulative distribution function of the raw scores must be determined on the basis of a sufficiently large and representative sample, which specifies this probability. The latter can, for example, be expressed in the form of percentiles. However, since percentiles do not represent a linear transformation of the raw scores, further computation with percentiles may lead to bias. Therefore, the percentiles are usually transformed into norm scores like, for example, *IQ*-scores or *z*-scores via normal rank transformation (i.e., by use of the inverse normal distribution function) and reported as such. The norming of psychometric tests can thus be defined as setting up population-based reference scores in order to be able to assess the exceptionality of an individual test result.

**Fig 1:**
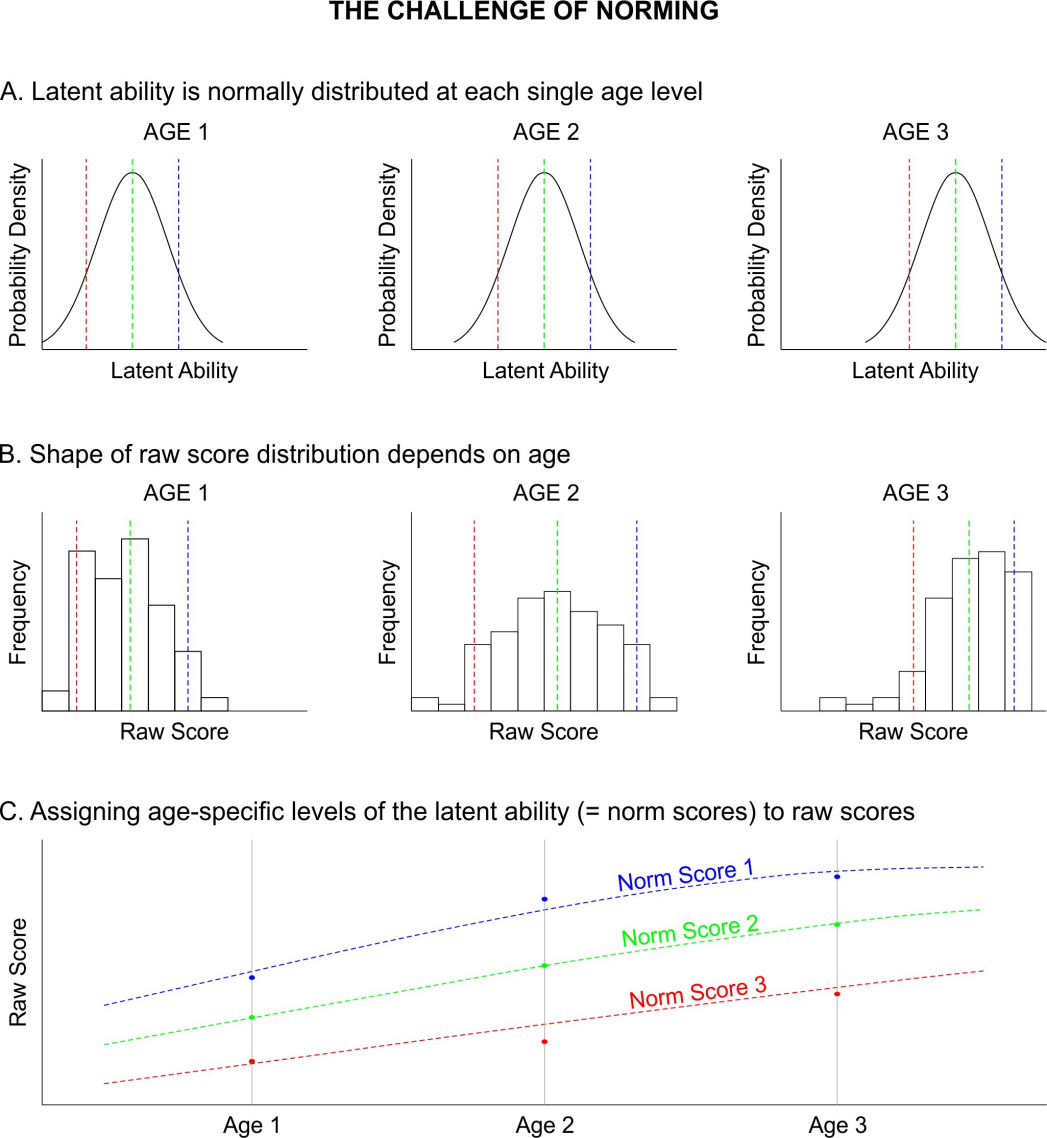
Norming as the group-specific (e.g., age-specific) mapping of raw scores to latent abilities. While the latent trait or ability is normally distributed in every single age group, the features and shapes of the resulting raw score distributions can change depending on age. As the norm scores are scaled with respect to each separate age-group, they reflect the exceptionality of the test result within a certain age group but do not reflect general developmental changes of the latent ability between age groups.

Many psychometric tests are based on the assumption that the raw scores are a manifest expression of a latent personality trait or ability which itself cannot be directly assessed. As highlighted in Fig 1, norming aims at mapping the raw scores of a test to that latent ability. While the latter one is usually assumed to be normally distributed, the same unfortunately does not apply to the raw score distribution. For example, most test scales have only a limited range of discrete possible outcomes, which can lead to skewed distributions constrained with floor or ceiling effects. The fact that abilities normally develop over the life span makes this process even more complicated. In intelligence tests, for example, the raw scores, which are supposed to reflect the level of the latent ability, increase swiftly with age. Therefore, a child has to be compared to a sample of children with the same or similar age in order to be able to assess the exceptionality of the individual test result. Consequently, in tests covering a wide age range, the normative samples have to be split up into multiple subsamples with a sufficiently narrow age range. This requirement quickly results in a large total sample size and can in addition lead to imbalances between the subsamples. At this point, continuous norming methods come into play. Their aim is to continuously model the change of the raw score distributions across age (as for example shown by the dashed lines in Fig 1C). Thus, norm scores can be specified for very narrow age brackets or even for specific age levels or schooling durations. Continuous norming can theoretically solve a whole series of problems of conventional norming: First, it reduces errors that occur due to random sampling. Second, it eliminates bias that occurs because a child's age does not correspond to the average age of the respective age group within the normative sample. Third, the norm tables do not contain the usual gaps of conventionally generated norm tables. Finally, continuous norming also requires smaller sample sizes than conventional norming and thus makes the norming procedures more cost-efficient [3–5].

There are several mathematical approaches, which can principally be used for continuous norming. They differ in at least three aspects, (1) the assumptions about the distribution of the raw scores of a test scale, (2) the optimization procedures implemented to increase the goodness of fit, and finally (3) the operationalization of independent variables and dependent measures.

A first way to create continuous norms consists in modeling the age progressions for each percentile individually, that is, the raw scores serve as the dependent variables, which are modeled as a function of age, while the percentile (i.e., the level of the latent trait or ability) is kept constant. This approach is also known as quantile regression [6,7]. It makes no specific assumptions about the raw score distributions and can therefore–in accordance with statistic convention–be classified as nonparametric. However, quantile regression is accompanied by some major disadvantages. First, individual percentile curves may intersect, which would violate the assumption that the assignment of raw scores to norm scores at a fixed age level must be monotonic. Second, as each percentile is modeled individually, the method is rather cumbersome and inefficient to apply within psychometrics. And third, error minimization or probability maximization of an overall model is not possible within this approach as there is no joint estimation or linkage between the individual percentiles. These reasons may have contributed to the fact that quantile regression has not been widely used to compute psychometric test norms. At least we do not know of a single test in which this method was applied. Therefore, although quantile regression may certainly be utile for the determination of specific cutoffs or for other particular psychometric purposes, we have nevertheless decided to concentrate in this article on those approaches that are actually in use for the computation of continuous test norms.

The most widely used approach, parametric continuous norming, has so far mainly been applied in intelligence tests such as the Wechsler series and KABC-II [8–14]. To our knowledge, the first test to introduce this kind of continuous norming was the WAIS-R [8]. This intelligence test for adults was launched in the U.S. in 1981. Its norming procedure is based on the assumption that the raw scores are normally distributed at each single age level [15]. Hence, the normative sample of the WAIS-R was split up into different age groups, for which the means and the standard deviations of the raw scores were computed separately. Subsequently, polynomial regression was used to model these two parameters as a function of age. As a result, the estimated mean and standard deviation of the raw score distribution could be determined easily from the regression equations for any specific age level within the normative sample. Finally, norm scores could be calculated for each raw score and age from these two parameters based on the normality assumption. However, violations of this assumption can severely bias norm scores generated with this method. Unfortunately, such violations frequently occur in psychometric tests. This drawback holds particularly true for tests that cover a wide age range, since floor and ceiling effects often occur at the boundaries of such an age range. Furthermore, in this relatively simple type of continuous norming, the means and standard deviations are modeled separately, which can lead to suboptimal data fit at individual age levels.

**Fig 2:**
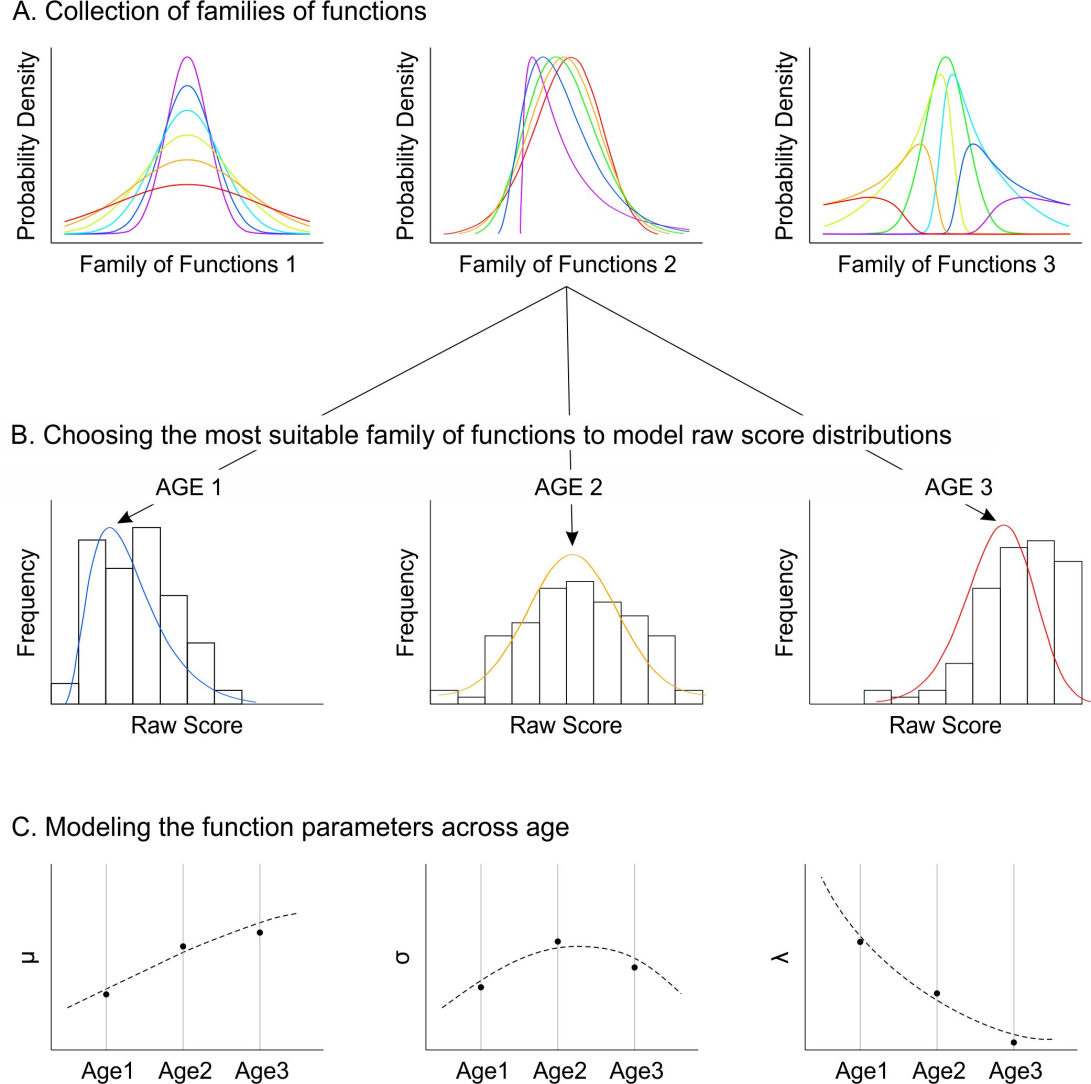
Parametric continuous norming. Known parametric functions are used to model the raw score distributions at specific age levels. The function parameters are subsequently modeled as a function of explanatory variables such as age.

To address violations of the normality assumption, researchers have suggested to first subject the raw scores to specific transformations aiming to approximate the raw score distribution to a normal distribution. For example, Cole and Green [16,17] used the so-called Box-Cox transformation for this purpose (see Fig 2A, family of functions 2). In addition to parameters for location and dispersion, the Box-Cox transformation has an additional skewness parameter λ, which can also be fitted across age. In further developments of this method [18–21], various families of functions have been used to transform the raw scores in such a way that the probability density of the resulting distribution is known. The functions vary in complexity, but generally possess three or more parameters capturing the location, dispersion, skewness and sometimes also the kurtosis of the raw score distribution. These parameters are used to approximate the probability densities of the raw score distributions at a fixed age. The age progression of the parameters is subsequently modeled with polynomial regression or related mathematical procedures (e.g., splines). In addition, the individual parameters are generally not fitted independently across age. Instead, joint likelihood functions are used, that is, the probability for the whole set of parameter values is maximized simultaneously. In line with the general statistical nomenclature, we summarize this type of norming procedure under the term *parametric norming* (Fig 2). Note that the specificity of this approach is not necessarily to use a specific number of parameters to model a distribution, but instead to use specific families of functions with characteristic shape (e.g., normal distribution, *t* distribution, exponential power distribution, beta-binomial distribution, SinH-ArcsinH distribution, and many more). For example, a function with only three parameters might fit a scale better than a function with four parameters when the typical shape of the three-parameter function is better suited for the specific raw score distribution. Therefore, when using parametric norming methods, the main psychometric task is to select the most suitable distribution function for a specific scale.

**Fig 3:**
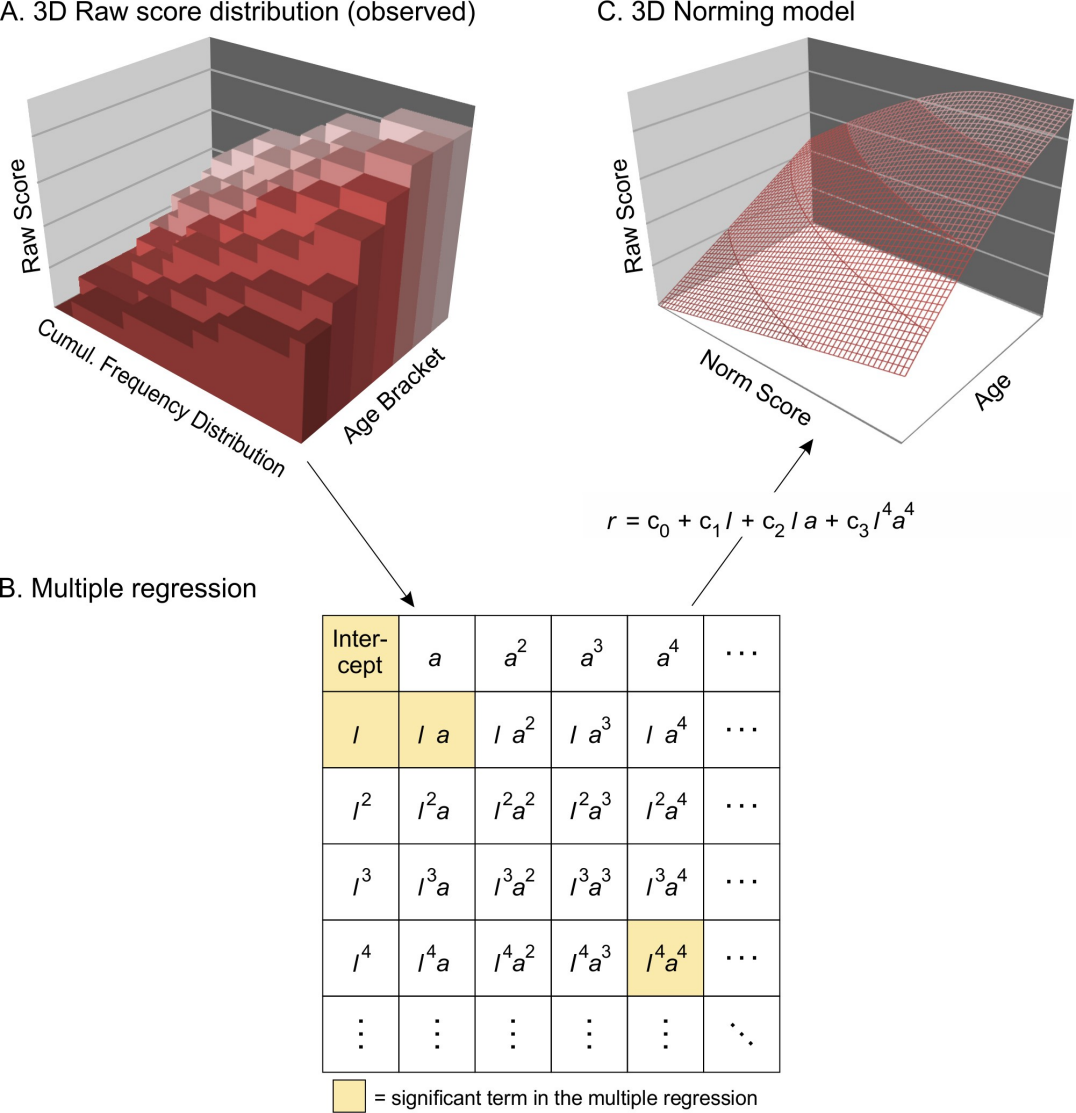
Semi-parametric continuous norming. Polynomial regression is applied to model a two-dimensional surface (age x location) in a three-dimensional space (age x location x raw score).

Our own approach [3,22,23] is a mixture between parametric and nonparametric methods and can therefore be characterized as *semi-parametric*. As with parametric norming, we use a type of parametrization to simultaneously fit the entire dataset. However, as with the nonparametric approach, we make no assumptions about the raw score distribution. Instead, we only assume that a raw score is the result of an interaction between the latent ability to be measured and the applied set of test items. Consequently, the dependent variable in our approach is the raw score–not the norm score. The age-specific person location *θ*_age_ (i.e., the level of the latent ability with regard to those persons of the reference population who have the same age) and the age *a* both serve as independent variables. As an estimator for *θ*_age_, we use the empirically observed percentiles, which are subjected to normal rank transformation prior to the modeling procedure (= observed location, *l*). The observed locations thus correspond to conventional norm scores. To fit the model, we apply polynomial regression. However, in contrast to the approaches described above, we do not fit curves, but a two-dimensional surface (age x location) in a three-dimensional space (age x location x raw score, Fig 3). The method can therefore be characterized as a regression-based fit of a hyperplane.

To better illustrate the rationale of this method, consider the optimal norming function *r*_exp_ = f(*θ*_age_, *a*). This function determines the expected raw score *r*_exp_, a person with location *θ*_age_ and age *a* is most likely to achieve on a given test scale. The problem with parametric methods is that even with the optimal parameter specification, the used families of functions are not necessarily able to adequately model the shape of this optimal norming function. For example, the normal distribution cannot model functions with skewness or kurtosis. Likewise, functions with a third parameter for skewness cannot account for kurtosis of the optimal norming function. Therefore, scales with large floor or ceiling effects are usually difficult to model with parametric distributions because they additionally include relevant higher-order moments (e.g., hyperskewness). In contrast, polynomial regression at least theoretically provides an error-free solution for any optimal norming function because an arbitrary set of *N* finite values can always be described mathematically by a polynomial of degree *N-1* as a function of another variable (or several other variables). Consequently, for a sample size of *N* = 1000, the entire range of expected raw scores could be modeled error-free with a polynomial of degree 999 as a function of person location and age. This means that the following equation would have to be resolved:

$$r=\sum_{s,t=0}^{999}{c_{st}\;l}^{s}a^{t},$$ (1)

which would, of course, be computationally complex. However, given that the raw scores of a test are normally limited to finite values, a conclusion is that the higher-order terms of the polynomial must quickly become very small and can therefore be neglected. Hence, only the first three or four powers of location *l* and age *a* usually have to be included into the regression equation to obtain sufficiently good data fit. The maximum power taken into account represents a smoothing parameter *k* of the norming function, that is, the lower *k* is selected, the smoother the function. The clipping of the polynomial also has the advantage to prevent data overfit. Finally, the optimal norming function is selected by including only terms that significantly improve the coefficient of determination in the regression equation [3,23]. Fortunately and in contrast to parametric continuous norming, the procedure does not require searching for a complex function to fit the data, because the polynomials will always provide a solution to the fitting problem.

#### A concrete example for applying semi-parametric continuous norming in test construction

R packages and in-depth tutorials exist both for the parametric [21,24] and for the semi-parametric approach [22,23]. Furthermore, a graphical user interface for the semi-parametric approach is available online via https://cnorm.shinyapps.io/cNORM/. With this interface, test constructors can model their data and assess whether the approach is adequate for their use case. To help readers, who are interested in applying semi-parametric continuous norming, we want to give a brief overview on the steps. In addition to the following demonstration, we advise readers to consult the online tutorial [22] for additional information and to repeat the procedure with the online user interface.

The procedure consists of the following steps:

1. Before statistical models are established, it is necessary to collect a sufficiently large normative sample and establish representativeness by stratifying it according to all relevant covariates (e.g., sex, schooling …). The online user interface includes an exemplary dataset on reading comprehension (‘elfe’ [25]) which includes seven age cohorts with the data of 200 children each. To our experience, smaller sample sizes (e.g., 50 cases per cohort, comp. [4,5]) already suffice to model the data, but establishing representativeness becomes increasingly difficult the smaller the dataset.
2. The next step is to prepare the data (tab “Preparation”) by computing conventional norm scores for the raw score variable, for example by applying a normal rank transformation or determining the percentiles within each age cohort. Afterwards, all powers of the norm score *l* (*l*, *l*^2^, … *l*^k^) and the age variable *a* (*a*, *a*^2^, … *a*^k^) plus the respective interactions (*l a*, *l a*^2^ … *l*^k^*a*^k^) must be computed up to the required degree *k*, as for example *k* = 4.
3. These variables subsequently enter a regression analysis as independent variables with the raw score serving as the dependent variable (tab “Modeling & Validation”). Finally, the significant terms of the polynomial and the respective coefficients are determined to set up the final regression formula [3]. The procedure usually leads to a simple norming models with only three or four significant terms in the regression and a coefficient of determination frequently above .98. It is advisable to keep the number of terms low, even at the expense of *R*^*2*^ in order to avoid overfitting.
4. Based on the regression function, the percentiles can be plotted (tab “Visualization”) to assess if the model fits the data. It might be necessary to adjust the number of terms in the regression, to rerun the modeling or to conduct a cross validation in order to retrieve optimal results. Finally, the model can be used for compiling norm tables or to directly retrieve norm scores for individual cases or datasets (tab “Prediction”). It is possible to assign norm scores to raw scores or vice versa as a function of age.

Unfortunately, semi-parametric norming–like quantile regression–can lead to intersecting percentile curves, which means that the monotonic relation between norm scores and raw scores at specific age levels is violated. Such inconsistencies most frequently occur when the test scale shows strong floor or ceiling effects (i.e., discriminating between different person locations on the basis of a limited range of raw scores is not possible). Therefore, violations of monotonicity can be used to select optimal models and to determine the valid measuring range [22,23]. In this study, we used them in a very simple way, which is described in the method section.

### Verifiability and transparency of norming procedures

As described in the previous section, there are several approaches available to set up continuous norming models. In contrast to other domains of test construction and analysis, quality criteria for evaluating these models are not sufficiently developed though. While statistical indicators for the reliability and validity of a specific test are usually reported in the respective test manuals to prove the test quality, one problem with continuous norming methods is that apart from a few exceptions known to us [26,27], the manuals of psychometric tests rarely provide evidence to prove the goodness of the norming model. Established procedures or specific characteristic values for this purpose are hardly available. Moreover, most test manuals even neglect to sufficiently describe the norming procedure detailed enough to allow for any statistical validation. In case of parametric continuous norming, they usually lack information about the chosen modeling functions. For example, it is not described whether or to which degree these functions fulfill the required distribution assumptions across the whole age range in the specific application. These shortcomings make it difficult for users to assess the limitations of the applied norming procedures and the resulting norm data. In our own efforts to fit psychometric test data on reading comprehension and vocabulary [26,27] with parametric norming methods, we particularly noted systematic deviations between the observed data and the fitted distribution curves at the lower and upper bounds of the raw score distributions and age ranges. The fact that these norming methods lead to biased norm scores especially in the extreme ability ranges, is all the more regrettable as these are the areas of the greatest importance in practical diagnostics [3].

Apart from the question on how to assess and communicate the goodness of fit of statistical norming models, there is also little information about which method provides the best modeling results under specific conditions. For example, the sample size, the distribution of item difficulties, the number of items within a scale and even the type of task or response format (e.g., power vs. speed test) could play a role when it comes to the selection of an optimal method. Since they have hitherto rarely been compared, it is impossible to choose the most suitable method in advance based on existing evidence. Instead, the selection of methods is limited to purely data-driven trial-and-error strategies, which can lead to suboptimal modeling results.

### Focus and rationale of research in this simulation study

The purpose of the present simulation study was to make a quantitative comparison of the different continuous norming methods in order to identify specific strengths and weaknesses under various conditions. However, comparing all methods under all possible conditions in one study is not possible. That is why in this article we focused on the two approaches we consider to be the most frequently used at present, namely parametric and semi-parametric continuous norming. In addition, we varied two general conditions that, in our opinion, are probably particularly important with regard to the goodness of fit of the respective methods, namely the sample size and the skewness of the raw score distributions. Variation of the latter was achieved through systematic changes of the average item difficulty.

As described above, the difficulty with parametric methods is to find a function and select a statistical model that fits the raw score distribution with sufficient precision, which is particularly challenging for raw score distributions with pronounced floor or ceiling effects. Therefore, our hypothesis was that the semi-parametric norming should outperform parametric norming in scales that do not provide sufficient items with very high or very low difficulty, that is, in scales that are generally very easy or very difficult for a given age. In addition, we suspected that deviations from an optimal norming model should manifest primarily in extreme ability ranges, because in these ranges the actual raw score distributions generally deviate most from the theoretical distribution assumptions.

A basic assumption of parametric continuous norming is that means, standard deviations, skewness and, where required, kurtosis of the raw score distributions can be estimated with relatively small sample sizes. For example, Zhu and Chen [5] stated that parametric norming with only 50 or 75 subjects per age group generally delivers sufficient goodness of fit. We therefore hypothesized that parametric continuous norming might outperform the semi-parametric approach when using small sample sizes.

## Method

### Data generation and statistical modeling

**Fig 4:**
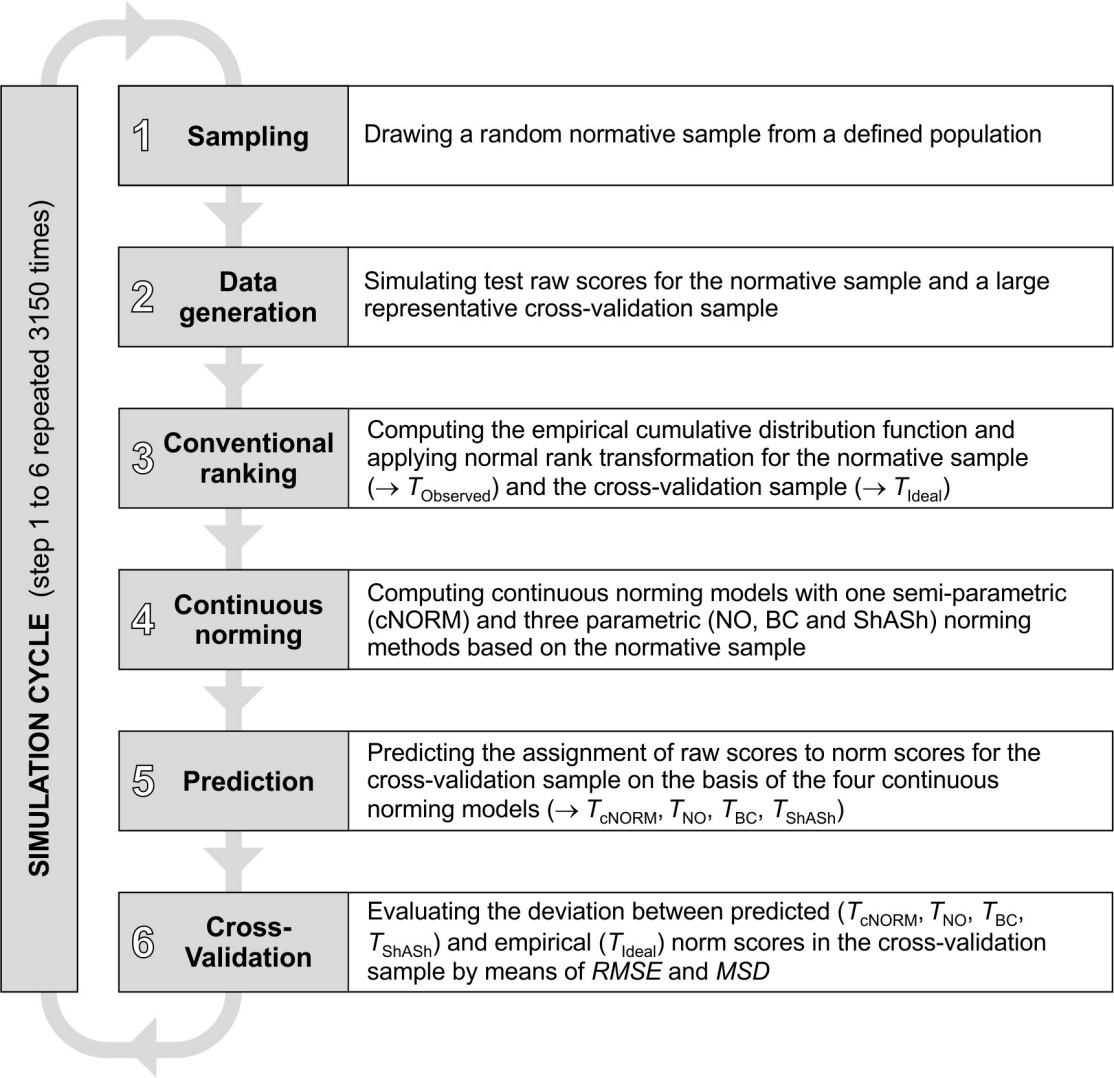
Flow chart of the simulation cycles.

In order to assess the goodness of fit of the different continuous norming methods, we first constructed a population model with defined developmental increase of a fictitious ability across age. We subsequently drew random samples of varying size out of this population and simulated a fixed number of item responses for each subject in these samples in accordance with a Rasch model. The average item difficulty of each set of items could be low, medium or high. The item responses were summed to a total test raw score and subjected to the different continuous norming methods. Finally, we evaluated the resulting assignments of raw scores to norm scores by use of a large and completely representative cross-validation sample. The six essential steps of the simulation are depicted in Fig 4. These steps were repeated 3,150 times.

Since we wanted to run the simulation cycles in an automatized way, we had to resort to methods that were both disclosed and available as software implementations, so that we could embed them flexibly in the simulation. We therefore used two open-source R packages in their latest versions: The GAMLSS (= Generalized Additive Models for Location, Scale and Shape) package [24] was applied to do the parametric modeling and the cNORM (= Continuous Norming) package [22,23] was used for the semi-parametric modeling (the packages are described in S1 Appendix; code and raw data is available through S1 Code and S1 Dataset).

The individual steps and features of the simulation are described in more detail below.

#### Population model

We constructed a developmental model of a fictitious intellectual ability for a population with the following seven age groups: age group 1 [0.5; 1.5[, age group 2 [1.5; 2.5[, age group 3 [2.5; 3.5 [… up to age group 7 [6.5; 7.5[. In addition to age, we assigned two person parameters to each subject. The first parameter, *θ*_Age_, functioned as a normally distributed and *z*-standardized person parameter, which represented the fictitious latent ability with respect to all subjects of exactly the same age. The second person parameter *θ*_Pop_, specified the latent ability in relation to the total population, that is, *θ*_Pop_ was *z*-standardized with regard to all seven age groups. *θ*_Pop_ was later used to simulate the test results based on the Rasch model (see Table 1 for an overview of statistical abbreviations and symbols). To model the development of the intellectual ability as realistically as possible, we used the normative sample of a real vocabulary test [26] as a blueprint. The following polynomials describe the mean and standard deviation of the unstandardized ability as a function of age:

1. *M*_*ability*_ = 1.5 ∙ Age—0.05 ∙ Age^2^ + 0.0001 ∙ Age^4^
2. *SD*_*ability*_ = 1 + 0.3 ∙ Age—0.01 ∙ Age^2^ + 0.00002 ∙ Age^4^
  The *z*-standardization of this ability across all seven age groups led to the following relation between *θ*_Pop_ and *θ*_Age_:
3. *θ*_Pop_ = (*θ*_Age_ ∙ *SD*_*ability*_ + *M*_*ability*_—5.097) / 3.128

**Table 1:** Statistical abbreviations and symbols used in the simulation.

Measure	Description
*θ*_Age_	Person parameter representing a latent ability that is *z*-standardized with regard to subjects of exactly the same age
*θ*_Pop_	Latent ability that is *z*-standardized with respect to the whole population instead of the specific age level of a person. This parameter was used to generate test data for the normative samples and the cross-validation sample.
*MSD*	Mean signed difference; measure to assess a general dislocation between predicted and ideal norm scores in the cross-validation sample
*RMSE*	Root mean square error; measure of the deviation between predicted and ideal norm scores in the cross-validation sample
*T*_Ideal_	Norm scores obtained from normal rank transformation of the empirical cumulative distribution function of the large and representative cross-validation sample. The raw scores assigned to these norm scores are essentially the raw scores statistically expected for a person with a certain latent ability and age.
*T*_cNORM_, *T*_NO_, *T*_BC_ and *T*_ShASh_	Norm scores predicted by the different continuous norming models (cNORM = semi-parametric; NO = Normal distribution family; BC = Box-Cox family; ShASh = SinH-ArcsinH family) on the basis of the normative samples

**Fig 5:**
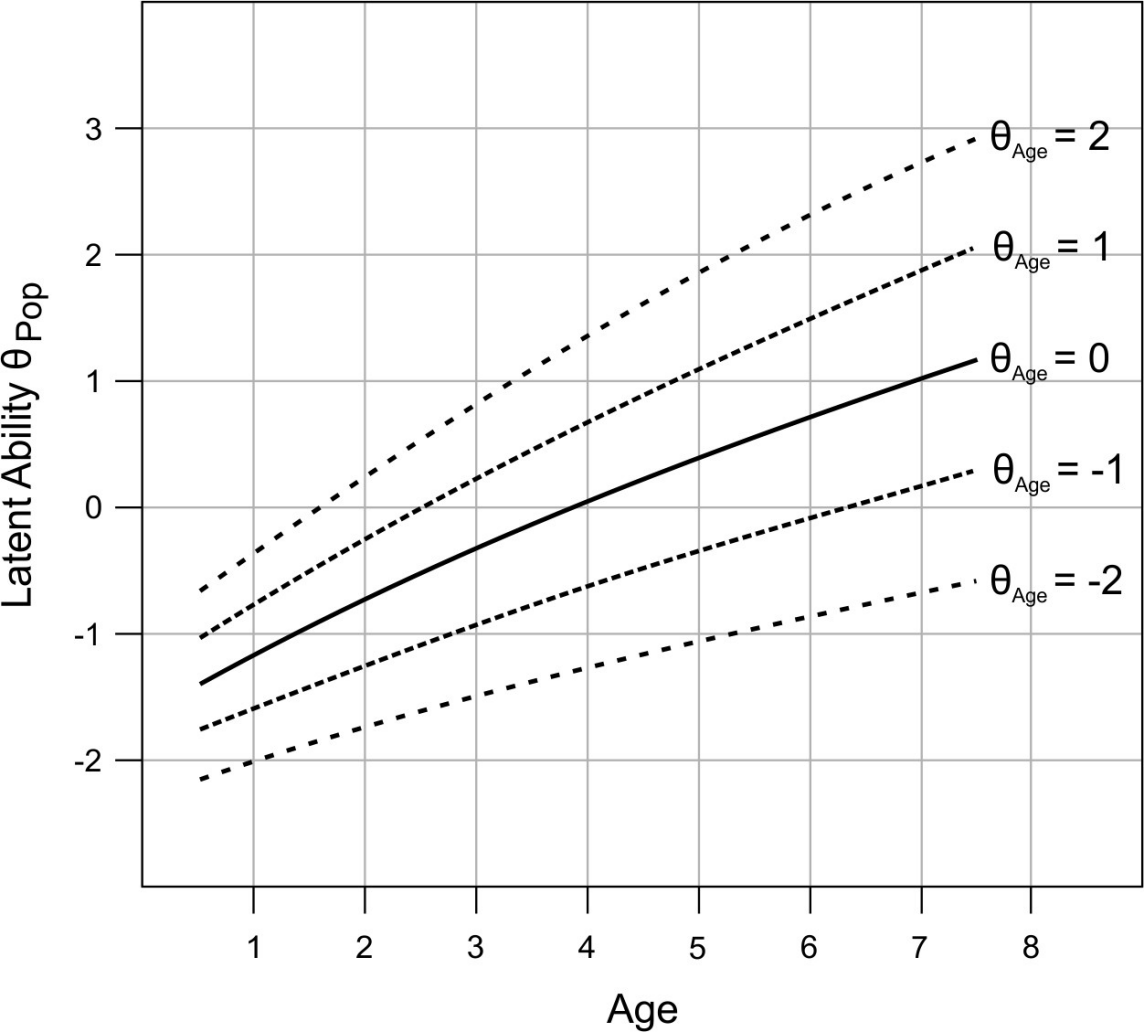
Age progression of the fictitious ability in the simulated population. *θ*_Age_ corresponds to the latent ability *z*-standardized with regard to subjects of exactly the same age and *θ*_Pop_ corresponds to the latent ability *z*-standardized with regard to the total population.

Fig 5 shows *θ*_Pop_ as a function of age. Note that due to the slightly curved increase across age, the latent ability parameter *θ*_Pop_ was not normally distributed within the total population but displayed a slight overall positive skew (*g*_*m*_ = 0.36).

#### Sampling

We generated normative samples according to the population model described above, that is, each sample contained seven age groups. Each age group had a size of *n* = 50, 75, 100, 150, 250, 500 or 1,000. We drew two random parameters for each subject in the samples. The first one was age, which was uniformly distributed across the specific age group. The second one was the age-specific latent ability *θ*_Age_, which was normally distributed with *m* = 0 and *sd* = 1. We additionally transformed *θ*_Age_ into *θ*_Pop_ as specified by the population model.

#### Simulation of test scales and test results

We generated three fictitious test scales with 20 test items each, following a Rasch model. We started the construction of these test scales with a scale of medium difficulty. In this scale, the item locations δ_i_ matched a perfect normal distribution with a mean value *M*_δ_ = 0.0 and standard deviation *SD*_δ_ = 1.0. As a result, the solution probabilities for the items were equally distributed between 0 and 1, that is, the items were optimally suited for the defined population in the sense that they covered the latent ability very well.

To create two additional scales with stronger floor or ceiling effects, we shifted the average item location to *M*_δ_ = -1.0 (easy scale) or *M*_δ_ = 1.0 (difficult scale) while keeping a standard deviation of *SD*_δ_ = 1.0 in each scale.

Because of the curvilinear trajectory of *θ*_Pop_, the skewness of the raw score distributions for the three different test scales varied in the individual age groups, with the medium test scale showing only a weak positive skew on average (*g*_*m*_ = 0.14), followed by the easy test scale, which had a moderate negative skew (*g*_*m*_ = -0.48). The difficult test scale showed the highest skew (*g*_*m*_ = 0.83) of the raw score distributions with a pronounced floor effect at the lowest age group. We therefore assumed that this scale should also entail the strongest differences with regard to the results of the different norming methods.

To simulate the raw score on one of these scales for a subject *i*, 20 item responses were simulated for this subject according to the Rasch model using the item difficulties of the respective scale. These responses were subsequently summed to a raw score *y*_*i*_ of this subject for the respective scale.

#### Statistical modeling

We subjected each normative sample to parametric and semi-parametric norming methods. The parametric modeling was performed with the *lms()* function of the GAMLSS package. We specified specific families of distributions with GAMLSS automatically choosing an optimal variant of the respective distribution, such as truncated or stretched. Our focus was originally on the Normal Distribution Family (NO, two parameters) and the Box-Cox Family (BC; including the three-parameter Box-Cox distribution sensu [16], the four-parameter Box-Cox Power Exponential distribution, and a truncated Box-Cox distribution). Since the BC family is not suitable for raw score distributions including zero (as is the case with the simulated data), we additionally used the SinH-ArcsinH distribution (ShASh) as recommended by the authors of the GAMLSS package [21]. Furthermore, to be able to still test the BC family when scores of zero where included in the raw score distributions, we simply added one point to each raw score when using the BC family for the modeling. This procedure is comparable to including a very simple test item in the test scale (i.e., an icebreaker item with a nearly 100% chance of being solved as is used in many psychometric tests). Such an item does not add information about the ability of a person to the test results and should therefore have no major impact on the modeling procedure (apart from the fact that the modeling is also possible with the BC family). This item was deducted when finally assigning raw scores to norm scores in order to be able to compare the assignments across methods.

For the semi-parametric modeling, we used the cNORM package with the default setting of *k* = 4 as maximum degree of the polynomial, which means that the resulting polynomial could contain up to 24 terms plus the intercept. In order to determine the optimal number of terms in the regression function, we used Ockham's razor, that is, a pragmatic law of parsimony stating that the simplest models should be preferred. Our previous experiments with modeling psychometric data had shown that, as a rule of thumb, usually the first four significant terms in the multiple regression are sufficient to set up a semi-parametric norming model with a coefficient of determination generally above .99. Our very simple selection algorithm was therefore to include four terms in the regression equation by default, and only to search for alternative models in case of violations of monotonicity in the resulting model. (Note that we only took into account violations within the range of the simulated normative data of each simulation cycle.) In case of violations, we started with three terms and consecutively added significant terms until a model without violations of monotonicity was found for the first time. This model was finally selected as the optimum model. If no such model was found, we used the maximum number of terms in the regression equation. In more than 75% of all simulations, this procedure resulted in a polynomial with only three or four terms for the entire norming model. In less than 0.1% of all cycles, the number of terms reached the maximum number of 24.

#### Cross-validation

The cross-validation sample contained only seven discrete age levels (i.e., 1.0; 2.0; 3.0; 4.0; 5.0; 6.0 and 7.0) with *n* = 10,000 subjects each, amounting to a total of *N* = 70,000 subjects. Furthermore, the person locations *θ*_Age_ in this sample matched a perfect normal distribution at each age level, making the sample perfectly representative at these specific levels.

The cross-validation was performed by generating a raw score of the respective test scale for each subject in the cross-validation sample. The generation of test data was performed in exactly the same way as in the normative samples. We subsequently computed conventional norm scores for each of the subjects. To this purpose, we subjected the empirical raw score distribution at each specific age level in the cross-validation sample to a normal rank transformation. Note that we will express these and all other norm scores in the results section in the form of *T-*scores with *M*_*T*_ = 50 and *SD*_*T*_ = 10. Given that each age level contained 10,000 subjects and had a perfectly representative distribution of the latent ability, we assumed that the resulting assignment of norm scores to raw scores represented a general upper limit of the norming quality of the respective test scale. We therefore refer to these norm scores as the *ideal norm* (*T*_Ideal_).

Subsequently, we converted the raw scores of each subject in the cross-validation sample into norm scores according to the four continuous norming models (parametric: NO family, BC family, ShASh; semi-parametric: cNORM) established on the basis of the much smaller normative samples. Consequently, five different norm scores were assigned to each subject in the cross-validation sample, namely the four modeled norm scores (*T*_cNORM_, *T*_NO_, *T*_BC_ and *T*_ShASh_) and an ideal norm score (*T*_Ideal_), which essentially corresponded to the norm score a person with a specific latent ability and age was statistically expected to achieve. An overview of the statistical abbreviations and symbols is given in Table 1. The norm scores generated with the continuous norming methods were subsequently compared to the ideal norm score with the methods described below.

We performed 150 cycles for each combination of sample size and scale difficulty. This resulted in a total of 7 x 3 x 150 = 3,150 simulation cycles. In some cases, the GAMLSS software failed to return a model for one of the parametric methods. In such cases, we restarted the cycle until a model was returned. cNORM never showed similar problems.

### Assessment of model fit

The simulation performed according to the procedure described above has the advantage that both the true age-specific location of each subject (*θ*_Age_) and the ideal norm score (*T*_Ideal_) for this subject are known. Note that *θ*_Age_ and *T*_Ideal_ do not match perfectly. Instead, *T*_Ideal_ cannot capture all variance of *θ*_Age_ because of the respective test-scale limitations (i.e., discrete raw scores, limited item numbers, suboptimal item difficulties and the non-linear development of the latent ability).

To compare the model fit of the different methods, we drew on the root mean square error (*RMSE*), which we determined from the difference between the norm scores predicted on the basis of the normative data (i.e., *T*_cNORM_, *T*_NO_, *T*_BC_ or *T*_ShASh_) and *T*_ideal_ in the cross-validation samples. Since *RMSE* is not normally distributed, we decided to count the number of simulation cycles in which each single method showed the lowest total *RMSE* of all methods. These data were subjected to *χ*^2^ tests on equidistribution. The significance level for all tests was set to *α* = .01 because of the risk of Type I errors with multiple comparisons. To assess the overall dislocation of the modeled norm scores from the norm scores, we additionally computed the *Mean Signed Difference* (*MSD*). As the analysis of the *MSD* was suggested in the review process of the article, we conducted a second simulation to obtain the according values. Therefore, the analysis of *MSD* and *RMSE* draws on two different datasets (available as S2 Dataset and S3 Dataset), but with otherwise identical parameters and equivalent results.

We also considered that most psychometricians would not mix both approaches in one norming procedure but would be more likely to choose either parametric or semi-parametric modeling. Furthermore, when using the parametric approach, they would probably compare different families of functions and subsequently choose the one that fits best (e.g., NO, BC or ShASh). To simulate this selection process, we additionally compared each single parametric method to one another and selected in each simulation cycle the one with the lowest *RMSE* as the best parametric method. Using this procedure it was possible to compare the two different approaches (parametric vs. semi-parametric) in general.

Finally, to be able to also analyze norming errors as a function of the latent ability, we segmented the measurement range (based on *T*_Ideal_) between the *T*-scores 20 and 80 (i.e., norm scores up to +/- 3 *SD*) into 12 intervals of five *T-*scores each and calculated the *RMSE* separately for these intervals. More extreme norm scores were excluded in this analysis because they usually play no major role in diagnostic decisions.

## Results

**Fig 6:**
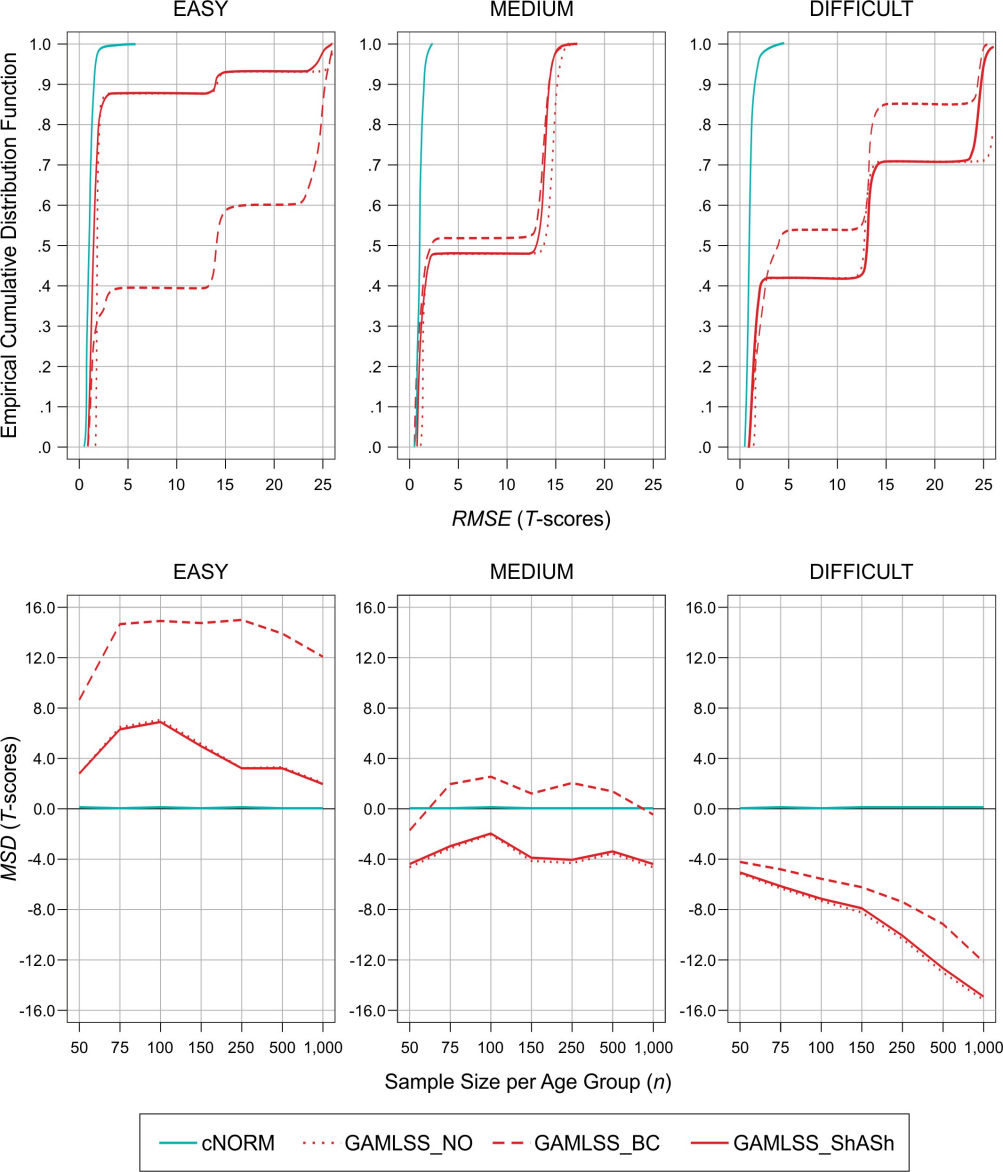
*RMSE* obtained by the different norming methods in the cross-validation sample as a function of scale difficulty (upper panel) and *MSD* per sample size and difficulty (lower panel).

Fig 6 shows the empirical cumulative distribution functions of the *RMSE* as a function of scale difficulty (upper panel). In this figure and all following figures, the turquoise line represents the semi-parametric method (cNORM) and the red lines represent the three parametric methods. The figure illustrates that the overall *RMSE* was below 2 *T*-score points in almost 98% of all simulation cycles, when cNORM was used to calculate norm scores. In contrast, the cumulative distribution functions of the *RMSE* for the parametric norming methods showed a cascade-like pattern, indicating that the GAMLSS software generated suboptimal results in a relevant number of simulation cycles. For example, applying the BC family to the easy scale led to more than 50% of simulation cycles with an *RMSE* above 10 (i.e., higher than 1 *SD* of the norm score distribution), which is a highly unacceptable value for any diagnostic purpose. Although the software had issued a warning message that the fitting algorithm did not converge in those cases, it had nevertheless returned a statistical model. The *MSD* (lower panel) illustrates that the parametric approach produced high systematic bias, which strongly depended on scale difficulty. The easy scale, which exhibits a pronounced bottom effect, led to a positive dislocation of the predicted scores in the parametric models and most pronounced in the Box Cox distribution family. The medium scale led to smaller systematic biases mostly in negative direction, with Box Cox being least affected. The difficult scale, which exhibits a ceiling effect, caused a large dislocation in negative direction in all parametric models with a considerable increase with sample size. cNORM in contrast was neither affected by sample size or scale difficulty and showed almost no systematic bias.

To give the parametric methods the benefit of doubt and to rule out biases related to missing convergence, we addressed this problem by assuming that an individual norming procedure with GAMLSS–in contrast to the simplified simulation procedure used in this study–would provide ways to avoid such severe mismatches without modifying the norming data (i.e., without eliminating alleged outliers). Therefore, to avoid an unfair bias due to the inclusion of obviously implausible models in the analyses, the descriptive data of *RMSE* and *MSD* subsequently reported in Table 2 are based on a cleaned dataset that only includes converged GAMLSS models with an *RMSE* below 10 (with cNORM, the *RMSE* was less than 10 in all cases). In this table, the row “Included” reports the percentage of models included in the analysis (i.e., with a total *RMSE* < 10).

**Table 2:** *RMSE* and *MSD* as functions of sample size *n* and scale difficulty in the cleaned dataset.

	Sample Size	50	75	100	150	250	500	1000	Overall
Scale difficulty: Easy									
**cNORM**	Included	100%	100%	100%	100%	100%	100%	100%	100%
	*RMSE*	1.412	1.257	1.197	1.077	1.001	0.913	0.772	1.090
	*MSD*	0.107 -0.055 -0.094 -0.054 -0.102 -0.064 -0.068 -0.078	0.055	0.094	0.054	0.102	0.064	0.068	0.078
**GAMLSS_NO**	Included	75.3%	78.0%	84.0%	96.7%	94.7%	89.3%	96.7%	87.8%
	*RMSE*	2.113	1.990	1.953	1.875	1.826	1.783	1.755	1.889
	*MSD*	0.269	0.154	0.245	0.185	0.234	0.219	0.219	0.219
**GAMLSS_BC**	Included	48.0%	43.3%	36.0%	40.7%	30.7%	35.3%	42.0%	39.4%
	*RMSE*	1.738	1.675	1.607	1.511	1.441	1.455	1.468	1.567
	*MSD*	0.177	0.106	0.281	0.112	0.161	0.147	0.204	0.171
**GAMLSS_ShASh**	Included	75.3%	78.0%	84.0%	96.7%	94.7%	89.3%	96.7%	87.8%
	*RMSE*	1.994	1.791	1.656	1.465	1.334	1.178	1.085	1.476
	*MSD*	0.349	0.253	0.336	0.204	0.247	0.196	0.131	0.242
**Scale difficulty: Medium**									
**cNORM**	Included	100%	100%	100%	100%	100%	100%	100%	100%
	*RMSE*	1.451	1.218	1.113	1.000	1.048	1.070	0.967	1.124
	*MSD*	0.037	0.038	0.083	0.026	0.055	0.034	0.039	0.045
**GAMLSS_NO**	Included	58.0%	45.4%	33.3%	28.7%	44.0%	54.0%	72.7%	48.0%
	*RMSE*	1.696	1.626	1.538	1.424	1.340	1.300	1.255	1.442
	*MSD*	0.112	0.074	0.124	0.085	0.110	0.114	0.082	0.100
**GAMLSS_BC**	Included	64.7%	50.7%	40.0%	34.7%	50.0%	56.7%	67.3%	52.0%
	*RMSE*	1.463	1.466	1.347	1.119	0.933	0.779	0.662	1.090
	*MSD*	0.151	0.095	0.170	0.133	0.124	0.136	0.117	0.133
**GAMLSS_ShASh**	Included	58.0%	45.3%	33.3%	28.7%	44.0%	54.0%	72.7%	48.0%
	*RMSE*	1.584	1.499	1.359	1.156	1.013	0.903	0.834	1.167
	*MSD*	0.131	0.081	0.134	0.087	0.092	0.102	0.064	0.099
**Scale difficulty: Difficult**									
**cNORM**	Included	100%	100%	100%	100%	100%	100%	100%	100%
	*RMSE*	1.558	1.194	1.147	1.024	0.908	0.795	0.752	1.054
	*MSD*	0.033	0.093	0.068	0.161	0.115	0.129	0.149	0.107
**GAMLSS_NO**	Included	59.3%	63.3%	56.0%	42.0%	32.7%	29.3%	10.7%	41.9%
	*RMSE*	1.867	1.759	1.678	1.599	1.554	1.495	1.473	1.6843
	*MSD*	0.100	0.193	0.092	0.101	0.039	0.038	0.062	0.103
**GAMLSS_BC**	Included	61.3%	68.7%	68.7%	64.0%	52.7%	40.0%	22.0%	53.9%
	*RMSE*	2.259	2.128	2.079	2.044	2.222	2.038	1.655	2.102
	*MSD*	0.021	0.032	-0.072	-0.050	-0.148	-0.084	-0.114	-0.044
**GAMLSS_ShASh**	Included	59.3%	63.3%	56.0%	42.0%	32.7%	29.3%	10.7%	41.9%
	*RMSE*	1.866	1.586	1.473	1.307	1.244	1.130	1.048	1.478
	*MSD*	0.211	0.322	0.226	0.255	0.172	0.158	0.194	0.233

Across all conditions, GAMLSS produced acceptable models in only 55.6% of all cases. Moreover, the proportion of acceptable models showed a complex pattern of sensitivity to sample size and scale difficulty. Interestingly, the analysis for medium difficulty revealed that the lowest proportion of acceptable models (30.7% across all three parametric methods) was found for a medium sample size of *n* = 150 per age group. A sample size of *n* = 100, which is the typical sample size used for continuous norming [8–14], yielded only a slightly higher proportion of acceptable models (35.5% across all three methods). For the scale with high difficulty, the proportion of acceptable models markedly decreased with growing sample size and was generally lower for NO and ShASh (e.g., only 10.7% acceptable models for *n* = 1,000) compared to BC. For the easy scale, the proportion showed a less uniform and less pronounced dependence on sample size but was generally lower for BC compared to NO and ShASh.

In line with our expectations, the semi-parametric method was by far the most preferable of all four methods when applied to the two suboptimal test scales, with the lowest *RMSE* in 87.9% of all simulation cycles when applied to the easy test scale, χ^2^(3) = 2,226.4, *p* < .001, and the lowest *RMSE* in 93.7% of all simulation cycles when applied to the difficult test scale, χ^2^(3) = 2,645.9, *p* < .001. However, contrary to our expectations, the semi-parametric method also showed the lowest *RMSE* in 64.0% of all cycles with the medium scale, χ^2^(3) = 1,057.8, *p* < .001.

The better performance of cNORM with regard to the suboptimal test scales was evident not only for the total dataset but also for the cleaned dataset (i.e., the data set including only models with *RMSE* < 10), with the lowest *RMSE* in 86.3% of all valid simulation cycles (i.e., simulation cycles with at least one parametric model with *RMSE* < 10) when applied to the easy test scale, χ^2^(3) = 1,861.1, *p* < .001, and the lowest *RMSE* in 88.4% of all valid simulation cycles when applied to the difficult test scale, χ^2^(3) = 1,217.3, *p* < .001. Moreover, in the vast majority of conditions, it exhibited the smallest *MSD* as well, while it has to be noted that in the cleaned data, there were only minor general dislocations in terms of *MSD* across all conditions and methods. Table 3 shows all pairwise comparisons within the complete (white cells) and the cleaned (grey cells) dataset as a function of scale difficulty.

**Table 3:** Pairwise comparisons between the methods in the cross-validation based on lowest *RMSE*.

Compared to:		cNORM	GAMLSS–NO	GAMLSS–BC	GAMLSS—ShASh
**Scale difficulty: Easy**					
Winner is cNORM	%		99.3	94.1	92.2
	*N*		1050	1050	1050
	*χ*^2^		1020.8***	816.8***	748.0***
Winner is GAMLSS–NO	%	0.8		57.4	6.6
	*N*	922		1050	1050
	*χ*^2^	892.7***		23.0***	780.2***
Winner is GAMLSS–BC	%	15.0	83.7		26.4
	*N*	414	412		1050
	*χ*^2^	202.9***	187.2***		233,9***
Winner is GAMLSS–ShASh	%	8.9	92.8	44.4	
	*N*	922	922	412	
	*χ*^2^	623.0***	675.6***	5.2*	
**Scale difficulty: Medium**					
Winner is cNORM	%		90.9	67.9	75.5
	*N*		1050	1050	1050
	*χ*^2^		702.6***	134.6***	273.1***
Winner is GAMLSS–NO	%	19.0		11.3	4.9
	*N*	504		1050	1050
	*χ*^2^	193.7***		629.0***	854.3***
Winner is GAMLSS–BC	%	61.7	90.6		67.8
	*N*	546	470		1050
	*χ*^2^	29.9***	309.9***		133.1***
Winner is GAMLSS–ShASh	%	51.0	90.1	20.0	
	*N*	504	503	470	
	*χ*^2^	0.2	323.5***	169.2***	
**Scale difficulty: Difficult**					
Winner is cNORM	%		97.9	95.9	96.1
	*N*		1050	1050	1050
	*χ*^2^		963.7***	884.9***	892.6***
Winner is GAMLSS–NO	%	5.0		47.5	33.9
	*N*	440		1050	1050
	*χ*^2^	356.4***		2.62	108.9***
Winner is GAMLSS–BC	%	7.6	47.4		49.1
	*N*	566	439		1050
	*χ*^2^	407.0***	1.2		0.34
Winner is GAMLSS–ShASh	%	9.3	84.8	64.0	
	*N*	440	440	439	
	*χ*^2^	291.5***	213.1***	34.4***	

Note. The % values indicate the proportion of valid simulation cycles in which the model in the leftmost column outperformed the compared model. Values in the white cells represent the complete dataset; values in the grey cells include only GAMLSS models with an *RMSE* < 10. All *df* = 1

* *p* < .05

*** *p* < .001.

**Fig 7:**
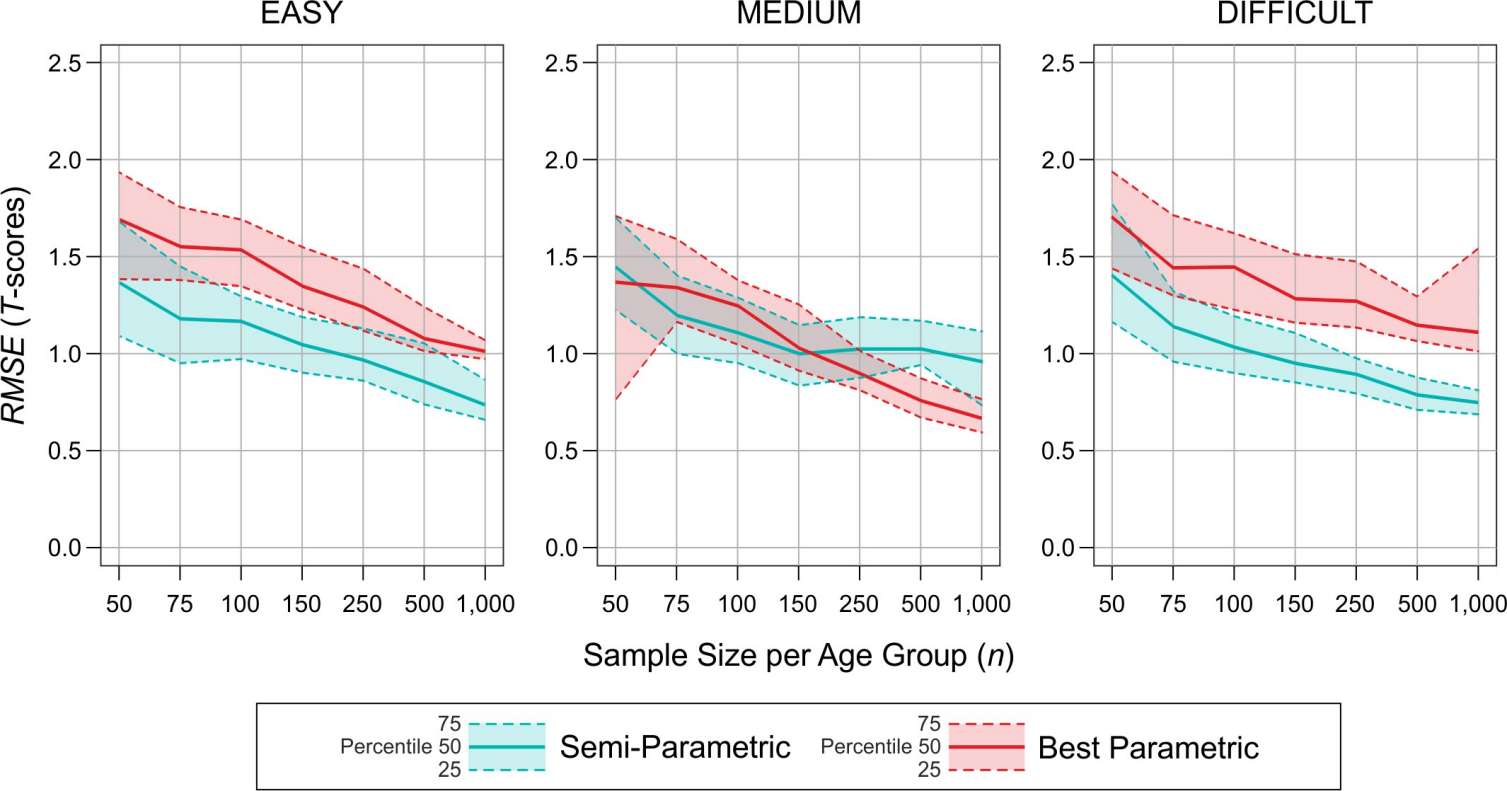
*RMSE* as a function of approach (semi-parametric vs. best parametric), sample size *n* and scale difficulty. The solid lines represent the 50th percentile, whereas the dashed lines represent the 25th resp. 75th percentile. This analysis includes all simulation cycles with at least one parametric model with an *RMSE* < 10.

It must also be noted, though, that after excluding all models with an *RMSE* ≥ 10, the general advantage of cNORM regarding the medium test scale vanished and was instead qualified by a threefold interaction between method, scale difficulty, and sample size. This effect is illustrated in Fig 7, which shows the *RMSE* as a function of approach (semi-parametric vs. best parametric), scale difficulty, and sample size. We included in this figure all simulation cycles returning at least one parametric model with a *RMSE* < 10. Given that the *RMSE* is not normally distributed, we chose to depict percentiles of the respective distributions in this figure instead of means and standard deviations. First, as can be seen from all three charts, the *RMSE* generally decreases with increasing sample size irrespective of the scale difficulty. Against our expectations, this pattern is similar for the semi-parametric and the parametric approach. Second, with regard to the medium test scale, the semi-parametric approach numerically performed best when the sample size was *n* = 75, 100 and 150 per age group. Yet, the counts yielded only a marginally significant difference for *n* = 75 (*p* = .07). Moreover, the parametric approach outperformed the semi-parametric when the sample size was *n* = 250 or higher (*p* < .001 for all tests). Note, however, that these sample sizes are only rarely used for continuous norming and that there was a selective dropout of the parametric models with increasing sample size. Third, in line with our hypotheses, the results produced with cNORM showed almost no dependence on scale difficulty, with an average *RMSE* of 1.1 for all three scale difficulties. In contrast, the results of the parametric approach were affected by scale difficulty, with the best results for the medium test scale (average *RMSE* = 1.0) and the worst results for the difficult test scale (i.e., the scale with the highest skewness; average *RMSE* = 1.5).

**Fig 8:**
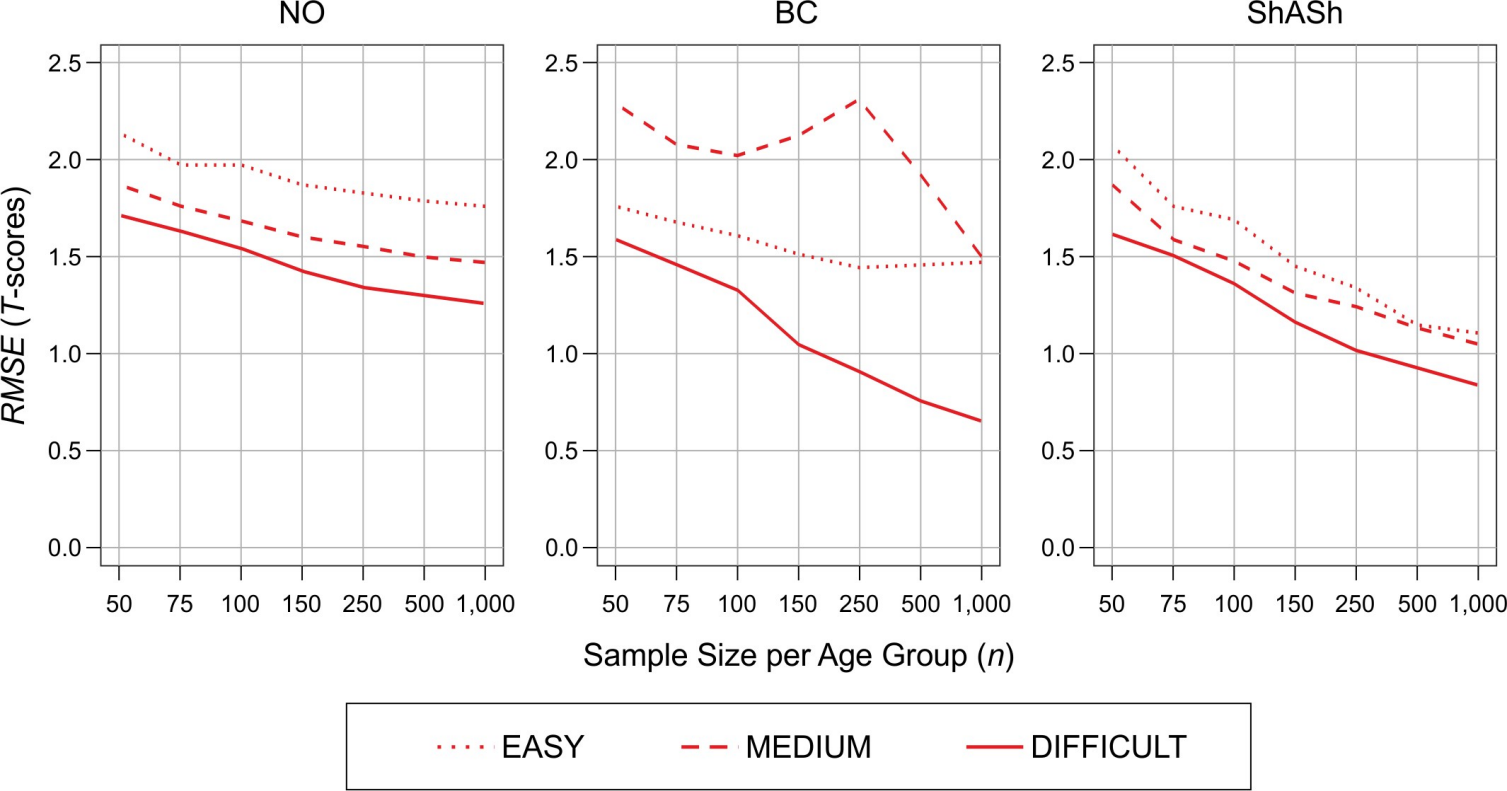
*RMSE* of the three parametric methods as a function of scale difficulty and sample size *n*. The figure includes all models with a *RMSE* < 10.

When focusing on the parametric approach only, the modeling results showed a pronounced interaction between the specific method and the scale difficulty (Fig 8). The dependence on scale difficulty was most pronounced for the BC family, which yielded by far the highest norming errors of all parametric methods when applied to the difficult test scale and the lowest when applied to the medium test scale. ShASh and NO also achieved the best results on the medium scale. However, the influence of skewness was much less pronounced for this scale, with ShASh showing the lowest sensitivity to skewness.

**Fig 9:**
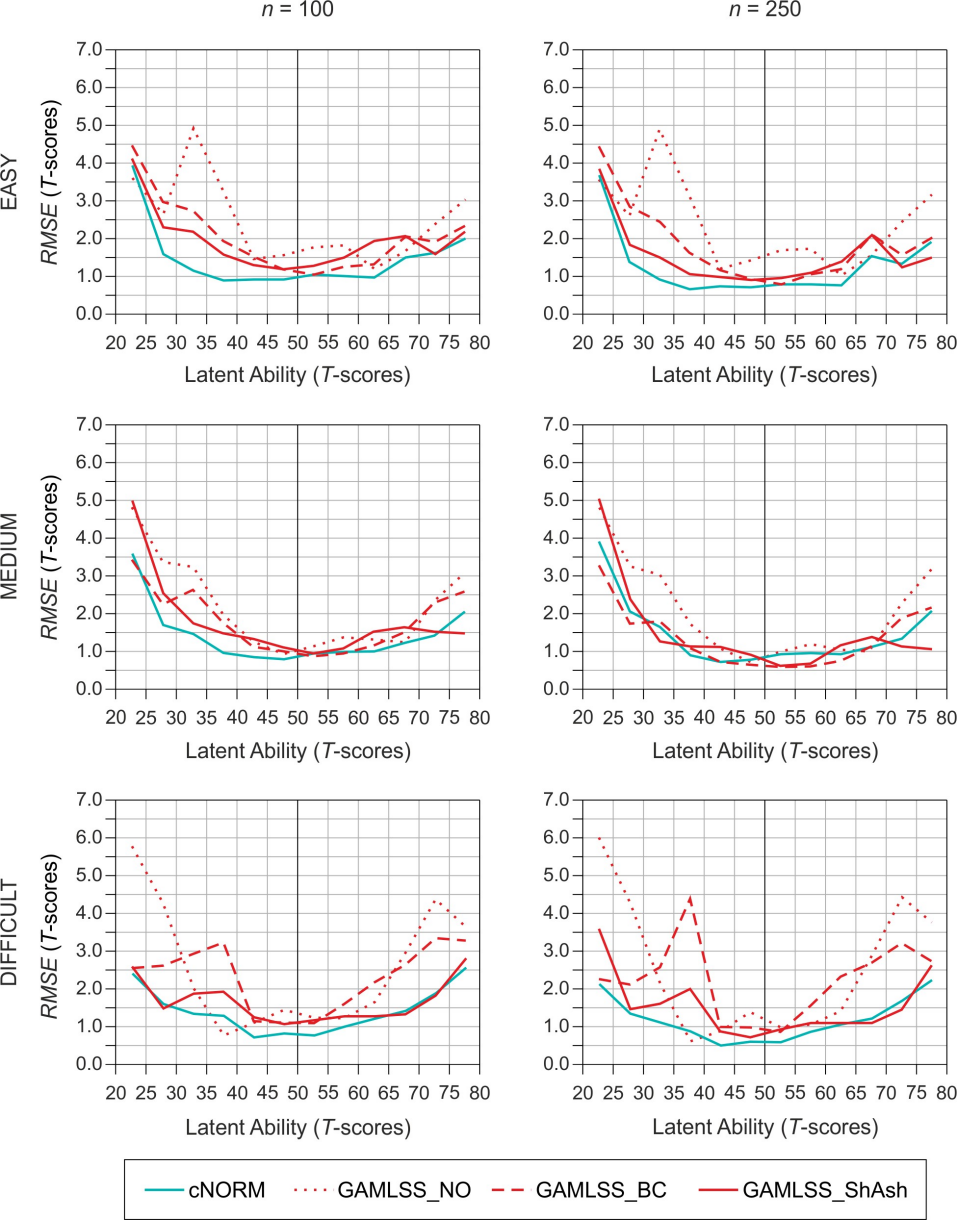
*RMSE* as a function of method, scale difficulty, and latent ability for sample size *n* = 100 (left panel) and *n* = 250 (right panel).

The relatively low average norming errors reported in the results above do not necessarily implicate superiority of a method across the whole ability range. Fig 9 shows the *RMSE* of the different methods as a function of scale difficulty and latent ability. The sample size of *n* = 100, which is typically used for continuous norming, is shown on the left panel. However, we also included a larger sample size of *n* = 250 (right panel), because BC as well as ShASh on average performed better than cNORM at this sample size and the size is still within the range of real-life application scenarios, whereas the next larger sample size of *n* = 500 per age group is used very rarely. First, as becomes clear when comparing all six charts depicted in Fig 9, cNORM very reliably delivered a U-shaped distribution of the *RMSE* as a function of ability independent of sample size and scale difficulty. Moreover, within a critical range of +/- 2 *SD*s around the population mean (i.e., *T*-scores 30–70), the *RMSE* never exceeded a *T*-score of 2. In contrast, *RMSE* showed obvious spikes at some ability levels when parametric methods were used. This pattern was especially true for the easy and the difficult test scale but also for the medium test scale with a sample size of *n* = 100. Furthermore, the location of the spikes barely depended on the sample size, which means that the deviations from the ideal norm scores are systematic. The analysis of the *MSD* also confirmed this result. (An analogous figure, depicting *MSD* as a function of method, scale difficulty, and latent ability, can be found in S1 Fig). The highest spike occurred between *T*-scores 30 and 35 when the NO family was used and was approximately 5 *T*-scores high (easy scale). This result was in line with our expectations that skewed distributions cannot be modeled adequately with a normal distribution. Noteworthy, however, was the fact that spikes of considerable size also occurred when using the BC family. For example, when applying BC to the difficult test scale, a spike occurred between *T*-score 35 and 40. Moreover, the spike clearly increased with sample size and was as high as 4.5 *T*-scores for *n* = 250, which strongly suggests that by using parametric methods, the norming error can significantly increase with sample size at individual locations even though it decreases on average across the whole ability range.

Only when BC or ShASh were applied to the medium test scale with a sample size of *n* = 250 no such spikes were detectable. Yet, neither BC nor ShASh outperformed cNORM at all ability levels, although both methods were slightly superior to cNORM on average. At *T* = 37.5, for example, cNORM showed the lowest *RMSE* of all methods. Hence, the overall differences between BC, ShASh and cNORM under this specific condition were so small that they would rarely have a significant impact on real test results, taking into account rounding accuracy.

## Discussion

### Summary of results

In this simulation study, we compared the reliability of different parametric and semi-parametric continuous norming methods depending on the skewness of the scale and the sample size. To this end, we repeatedly drew random normative samples with varying size and generated test results for these samples based on a Rasch model. We used test scales with three levels of difficulty, namely, a scale with an optimal distribution of item difficulties (medium scale), a scale with a moderate ceiling effect (easy scale), and a scale with a pronounced floor effect (difficult scale). We subsequently generated statistical norming models from the test results according to the different methods and assessed the model fit with a large and completely representative cross-validation sample.

The semi-parametric approach outperformed the parametric one under most conditions. In line with our expectations, semi-parametric norming yielded significantly lower norming error for the suboptimal test scales with floor or ceiling effects. This superiority was independent of the sample size. The largest differences between the semi-parametric and the parametric approach emerged for the test scale with the highest skewness of the raw score distribution (i.e., the difficult test scale). Note that the main problem is not necessarily the skewness of the data but rather the floor effect. However, given that real psychometric test scales usually have a very limited number of items and that the distribution of item difficulties often is far from perfect, floor and ceiling effects are, in our experience, not the exception but rather the rule.

For the test scale with medium difficulty, the overall performance of both approaches was more balanced. At those sample sizes typically used for continuous norming, the semi-parametric method yielded slightly better results than the best fitting parametric method, while for higher sample sizes it was exactly the opposite.

It was also noteworthy that the quality of the three parametric methods depended to different degrees on the scale difficulty. While the norming error only slightly depended on the scale difficulty when using the ShASh function, the BC family yielded the lowest average norming error of all parametric methods when applied to the medium test scale and the highest when applied to the difficult test scale.

The analysis of the norming error as a function of the latent ability yielded even more detailed results. As expected, the norming error reliably showed a U-shaped distribution when using the semi-parametric method, regardless of sample size and scale difficulty. By contrast, applying the parametric methods resulted in considerable deviations from the ideal norm at specific levels of the latent ability, that is, the individual norming error was low at some ability levels but very high at others. Large deviations of the ideal norm occurred not only in the extreme ability ranges, but also in the moderately above- or below-average ranges. In some cases, the height of the observed spikes even increased with growing sample size. Although the magnitude of the effect depended on the skewness of the scale and the specific parametric function, the same pattern was observable for all three parametric families of functions. Specifically, the spikes were unexpectedly high for the BC family when applied to the difficult test scale. In fact, Cole and Green [16] stated in their original article that the BC function is only suitable for moderate skewness. Nevertheless, we had expected at least a slight performance advantage of the BC family over the NO family for the difficult scale, because when the NO family is used, skewness can only be modeled by truncating the distribution, which only allows a very rough adjustment. Moreover, the norming error depicted in Fig 9 was averaged across all age groups, which means that the spikes must have been even higher at specific age groups. Under unfavorable conditions (e. g., specific combinations of age and ability level), parametric norming can therefore yield individual norming errors of more than half a standard deviation, even if the specific method shows low norming error when averaged across all age levels and abilities. Such deviations from the ideal norm score could, of course, lead to serious diagnostic mistakes.

At this point, it is important to raise the question of how a test author or psychometrician would handle marked deviations from the empirical data at specific ability levels or age groups. The first step in addressing deviations is to detect them. An urgent recommendation when applying continuous norming methods is therefore to visualize and inspect the model fit thoroughly over the entire ability and age range. We will revisit this requirement later. The second step is to interpret the deviations as errors of the norming model and not as outliers or random noise of the empirical data. We are concerned that deviations are often misattributed to the data instead of the statistical model, with the latter being the only valid interpretation in our simulation. In line with this apprehension, one of the authors of the GAMLSS software recommends reducing detectable spikes by eliminating supposed outliers [28]. This procedure would not have reduced the norming errors in the present study but instead would have overestimated the model fit. We therefore suggest to apply this strategy cautiously and sparingly.

Our last hypothesis, which predicted that the semi-parametric method would be more sensitive to a change in sample size than the parametric methods, was not supported. Both approaches benefited from an increase in sample size to about the same extent. It has to be noted, though, that a substantial increase in sample size would in most cases only lead to minor improvements in the precision of individual norm scores. Therefore, the results are still in line with the assumption that continuous norming generally can draw on smaller sample sizes compared to traditional norming procedures to achieve comparable results [4,5]. Moreover, as described above, when using parametric methods, convergence problems and even the deviations from the ideal norm at specific ability levels grow with larger samples. This finding might, in our opinion, result from an increase in statistical power. Given that the parametric modeling to some extent forces the raw score distributions into unsuitable shapes, the overall error can only be kept low when high error is tolerated in some locations. This feature of parametric modeling leads to the paradoxical effect that an increase in sample size might as well increase the bias of the modeled norm scores.

Nevertheless, it must also be taken into account that the smaller the sample size, the more difficult it is to establish sufficient representativeness of the normative sample in real life applications. This is especially true if several stratification variables must be taken into account. Based on our own experience, the usual sample size of *n* = 100 per age group used for continuous norming can often place enormous demands on the balancing of the most important stratification variables (e.g., age, sex, ethnic group, geographic region etc.). Hence, the combination of semi-parametric norming with moderately large representative samples (e.g., *n* = 100 to 200) seems to be the most solid approach to psychometric test norming in most application cases, while at the same time being sufficiently cost-effective.

### Limitations of the study

One problem we had to deal with in this simulation study was the relatively low percentage of valid models returned by the software used for the parametric approach. Only about half of the parametric models showed satisfactory model fit. In the other half, the used algorithms seemingly did not converge and therefore returned models with unacceptable model fit. This problem even occurred for the test scale with optimal item difficulties, for which we had expected the parametric approach to outperform the semi-parametric one. In our study, we could easily handle this problem by running a sufficiently large number of simulation cycles and including only the converging models in the subsequent analysis. In applied settings, however, psychometricians must analyze only one ‒ often imperfect ‒ dataset. The question therefore is how they should address the problem of non-convergence in real-life applications. In many of our simulation cycles, only one of the applied families of functions failed to yield a converging model, whereas the other models converged. Since we chose the best parametric method only from the converging models, we might have selected suboptimal models in some cases, because perhaps a function that failed to produce a converging solution in a specific case could theoretically have been better suited for the data. Most psychometricians would probably choose the appropriate function in a similar way, that is, based on the lowest *RMSE* between empirical and modeled data, when applying the parametric approach in practice. Theoretically, it may be more advisable to select the function or family of functions on the basis of the specific data properties (e.g., skewness, kurtosis, sample size, floor and ceiling effects and so on) in advance, rather than adopting a trial-and-error strategy. If, however, the pre-selected function does not return a converging model and the psychometrician meets this problem by sorting out supposed outliers from the normative data, this procedure can again lead to suboptimal models. It is therefore difficult to assess the practical impact of this problem.

Another shortcoming was that in the present study we could not evaluate all available parametric functions but had to pre-select three specific families of functions. We focused on these functions, because they are used in real psychometric tests [29] or were recommended by the authors of GAMLSS [20] under specific conditions. We documented strengths and weaknesses of these families of functions depending on the respective properties of the simulated datasets. The GAMLSS package offers numerous additional functions to model raw score distributions, which might fit our simulated datasets even better than the functions we used. To the best of our knowledge, however, no further simulation studies are available to date in which the goodness of fit of these functions has been demonstrated for scales with specific properties. Consequently, a psychometrician applying the parametric approach in practice would again have no choice but to determine the function on the basis of a pure trial-and-error strategy, which we have already rejected as suboptimal.

There are a few other conditions that may possibly affect the goodness of fit of the evaluated methods, we could not systematically vary in this single study. For example, we used a fixed number of items in the test scales. In practice, a higher number of items could reduce the skewness of a scale, but only if the item difficulties were equally distributed across the whole ability range. Reducing the skewness would be especially beneficial for parametric norming with the BC family. As a consequence, differences between semi-parametric and parametric norming could be partly narrowed. However, as already stated above, a perfect equidistribution of item difficulties across the whole ability range is no realistic scenario, because very easy or very difficult items tend to provide unfavorable item parameters and therefore are often eliminated during test construction.

In addition, we based our simulation on a Rasch model, which is only applicable for pure power tests. One task of future simulation studies would therefore be to check the fit of different norming methods for simulated data with more complex measurement models including speed as well as power components.

Another problem of the simulation study was the necessity to rely on default settings of the respective software packages instead of individual adjustments of parameters as would be the case in applied settings. For example, we used a default smoothing algorithm and an automated model selection within each family of functions when applying the GAMLSS package. Manually selecting the respective parameters might have led to better modeling results. On the other hand, the selection strategy used with cNORM was also extremely simple and far from optimum. It is therefore difficult to assess to what extent an individualized adjustment of the modeling parameters would have influenced the results of this study.

**Fig 10:**
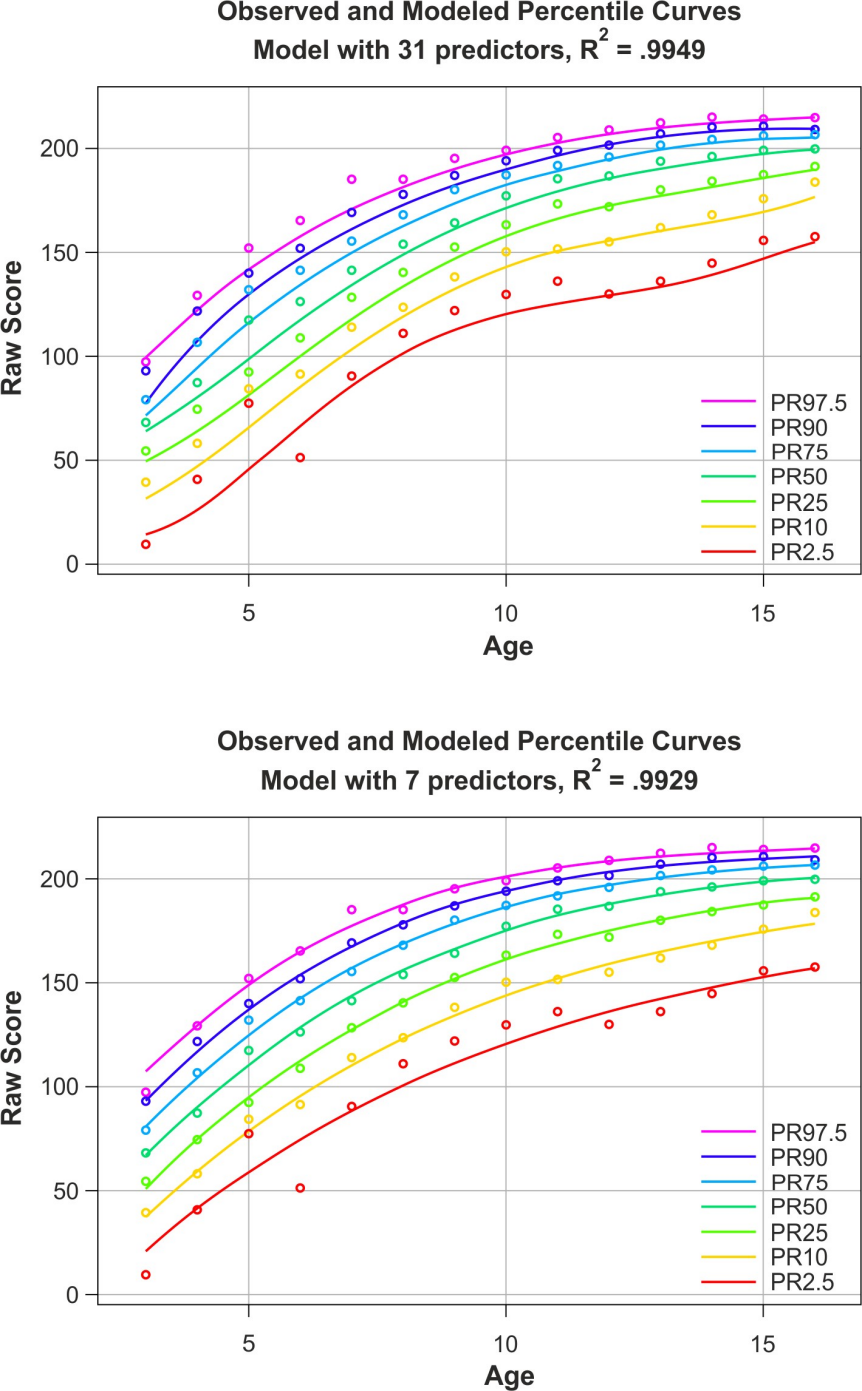
Percentile curves generated with the cNORM package based on the normative sample of a real vocabulary test. The curves show which raw score (y-axis) is assigned to a specific ability level (each represented by a percentile curve) at a certain age (x-axis). The upper panel shows a deliberately ill-executed norming procedure (31 terms, *k* = 5, raw score *RMSE* = 3.33, norm score *SE* = 0.88), whereas the lower panel depicts an optimally executed procedure (7 terms, *k* = 4, raw score *RMSE* = 3.83, norm score *SE* = 1.11).

A final limitation of this study is that we did not compare continuous norming to conventional norming. A major reason for this shortcoming is that we wanted to evaluate the continuous norming models at discrete age levels and therefore tailored the cross-validation sample accordingly. However, a major source of error in the application of conventional norming is precisely the fact that the norm scores are not provided for any specific age level, but for large age ranges, with some children being relatively far from the average age of the respective age group. We would not have been able to capture this source of error with the specific design of our study, that is, it would have been biased in favor of conventional norming. Based on our previous experience, we can, however, state that continuous norming generally leads to lower bias of the norm scores when a suitable method is selected and carefully implemented [3]. Fig 10 (lower panel) illustrates this assumption by showing the relation between continuous and discrete norming. The data on which this figure is based were obtained from a real vocabulary test [26]. The conventional norms (depicted as dots) show considerable variation between the individual age groups. A heavily jagged age course is discernible particularly in the ability range, which is strongly below average (PR 2.5). It seems very unlikely that such an irregular pattern would actually reflect the true age progression of a latent ability. In contrast, the continuous norming method (depicted as lines) compensates for the large differences between the individual age groups and provides smooth percentiles across the entire ability range.

### Recommendations for the evaluation and documentation of continuous norming procedures

Although continuous norming methods have been in use for several decades, few peer-reviewed articles have been published on this topic to date. Moreover, to the best of our knowledge, not a single textbook on psychometrics contains any information about these methods. Even more concerning, however, is the fact that the test manuals themselves also rarely provide sufficient information about the continuous norming methods and the goodness of fit of the resulting models. As a first step to address this problem, we complete our article with a few concrete recommendations for quality assessment and documentation of continuous norming procedures.

#### Quantitative criteria

In our simulation study, we quantified the fit of the different norming methods with *RMSE* and *MSD*. The first is commonly used to evaluate the goodness of fit of statistical models, because it captures all kinds of global and local differences between observed and modeled data and can be applied very flexibly. In our specific case, we used the *RMSE* to compare observed distributions based on simulated data of an ideal sample with modeled distributions based on simulated data of suboptimal samples. Thus it was possible, to analyze the extent to which the quality of the norm scores generally varied depending on the sample size, the skewness of the raw score distributions, the ability level and the norming method. *MSD* on the other hand was used to assess a general dislocation of the fitted to the observed scores. Other researchers in this field have suggested calculating confidence intervals for norm scores based on repeated sampling [4,30].

In general, we would like to encourage test authors to generally report more descriptive statistics that play or could play a role in the assessment of the norming procedures. For example, we have demonstrated in this study that the skewness of the raw score distribution (resp. the presence of floor or ceiling effects) is an important criterion with regard to the selection of suitable parametric norming functions. Skewness parameters of the raw score distributions could be reported very easily in test manuals.

#### Qualitative criteria

Another very simple way to document the quality of the norming models in the test manuals is to provide graphical illustrations of the empirical data and the modeled percentile curves across the entire age and ability range. Contrary to quantitative criteria, the model fit can be assessed intuitively and with only minimum prior knowledge with such illustrations. Compare, for example, the upper and the lower panel of Fig 10, which both depict percentiles modeled with cNORM based on the same norming data of a real vocabulary test. The upper panel shows percentiles we deliberately generated in a negligent and therefore suboptimal way. The lowest percentile curve in this panel (PR 2.5) shows a fairly curvy trajectory, which contradicts theoretical assumptions about the monotony of the vocabulary growth during adolescence. The observed development of the raw scores across age was most likely overfitted in this case. In contrast, the lower panel depicts diligent modeling of the same data set (which, by the way, was based on an extremely simple mathematical model). The coefficient of determination *R*^2^ is slightly lower than in the upper panel, but the percentiles are smooth and increase monotonously. It can therefore be assumed that a good model fit has been achieved without overfitting the data, which could further be verified by cross validating the model.

#### Concluding remarks and outlook

In the field of continuous norming, the scientific discourse on the comparison of different norming methods is largely lacking. Only in the last few years, open source software for that purpose is available to test authors and other scientists in the form of R-packages. Although the mathematical methods and functions are fully disclosed in these software packages, the evaluation and application of the specific continuous norming methods and software packages may still be a major challenge for many test authors. We will gladly provide assistance with the application of our own method and software package, and we have also experienced comparable help with the authors of the GAMLSS package, whom we would like to express our thanks to on this occasion. Both packages provide measures to quantitatively and qualitatively assess and document the quality of the produced norm scores and are therefore very useful tools for improving psychometric tests.

The aim of our work is to provide a more precise assessment of human abilities and achievements and thus also to improve decision-making in the field of applied psychometrics. We hope that with this study we have taken another small step towards achieving this goal.

## Supporting information

S1 Appendix Description of the technical aspects of the packages GAMLSS and cNORM. (DOCX)

S1 Code R code for conduction the simulation. (R)

S1 Dataset Raw data including the validation sample and the item parameters for usage in the simulation. (RDA)

S2 Dataset Results of the simulation study. (SAV)

S3 Dataset Replication of the results with additional information on *Mean Signed Difference* (*MSD*). (SAV)

S1 Fig *Mean Signed Difference* (*MSD*) per ability level in dependence of method, scale difficulty and sample size. (TIF)
