# Supplementary material for: Continuous norming of psychometric tests: A simulation study of parametric and semi-parametric approaches
Source: PLoS One. 2019 Sep 17;14(9):e0222279. doi: 10.1371/journal.pone.0222279 (PMC6748442; doi:10.1371/journal.pone.0222279)
Supplement: S1 Appendix — (DOCX) [file pone.0222279.s001.docx]

### Support material S1 – Description of the packages

The simulation study is based on the following two open-source packages, available via the R platform. Both have the advantage that their source code is fully disclosed. Although some of the methods might be too complex to be described in full length in an article or a test manual, they thus can at least in principle be retraced in detail if desired (and referred to in test manuals).

### GAMLSS

The GAMLSS package[1] has implemented a plethora of different families of functions which can be used for parametric continuous norming. The selection of the most suitable function is either performed automatically via an optimization algorithm of the software, or it can be applied manually. The modeling of the distribution parameters is performed with a joint likelihood function, that is, the likelihood is not optimized separately for each individual parameter, but simultaneously for a specific parameter constellation.

The age progression is typically modeled with so-called B-splines. In this method, the curves are split up into smaller pieces (i.e., small age intervals), and a polynomial regression is performed for each piece. These single segments are finally reassembled. B-splines are typically used to model very curvy trajectories. However, this procedure also entails the risk of overfitting the data. Therefore, GAMLSS contains different methods to minimize the Generalized Akaike Information Criterion (GAIC). This purpose of this index is to establish an optimal relation between goodness of fit and the number of estimators required. The user can specify the optimization method manually. To avoid over-fitting in the simulation study, we performed a pilot study of 100 cycles to assess the difference between the different smoothing methods implemented in GAMLSS (i.e. the local maximum likelihood optimization functions *ML()* and *GAIC()* (together with a recommended smoothing parameter of κ = 3[3 p454]). The application of the different smoothing methods was balanced across all conditions. Our data analysis revealed no discernable advantage for either method. Thus, we used the default ML optimization in the main study. We used the default setting for all other parameters of the modeling.

### cNORM

The cNORM package[3,4] is available as an open source project and contains only one type of semi-parametric continuous norming, namely the regression-based modeling of a hyperplane described above. For polynomial regression, cNORM uses the regsubsets method of the leaps package[5] (selection algorithm based on Miller[6]) in R to select the best models. To avoid overfitting, cNORM offers the possibility to vary the degree of the polynomials with lower degrees, which produces smoother curves. In addition, for a given degree of the polynomial, the number of terms to be included in the regression equation can be specified. Based on our experience from test construction projects, a degree of 4 is usually both adequate and necessary to sufficiently model the data and is therefore used as the default value in cNORM.

In contrast to parametric continuous norming, the raw score is modeled as a function of the norm score. This approach requires that the resulting polynomial must finally be resolved according to the person location to find the appropriate norm score to a given raw score and age. To address this requirement, we use determined the zeros of the age specific regression function using the *polyroot* function of the stats package in R (based on Jenkins and Traub[7]). In the case of multiple solutions, we searched for the most plausible solution based on a numerical strategy (*optimize* function, based on Brent[8]).

In the modeling, we as well used cNORM for assigning manifest norm scores to the raw scores. For the norm samples, the estimation of the age-specific person location of a subject *i* was performed with a sliding window of size 1 with the *rankBySlidingWindow()* function. A setting of 1 means that the normal rank of each subject is determined in relation to all subjects whose age lies within an interval of size 1 around the age of subject *i* [*age_i_* - 0.5; *age_i_* + 0.5]. The ranking of the validation sample was done with the *rankByGroup()* function, because here, only discrete groups were used.

### Literature

1. Stasinopoulos DM, Rigby RA, Voudouris V, Akantziliotou C, Enea M, Kiose D. Package ‘gamlss’ (Version 5.1‒2). Cran-Repository; 2018Oct06.
2. Stasinopoulos DM, Rigby RA, Heller GZ, Voudouris V, De Bastiani F. Flexible Regression and Smoothing – Using GAMLSS in R. Boca Raton: CRC Press; 2017.
3. Lenhard A, Lenhard W, Gary S. cNORM - generating continuous test norms [document on the internet]. Dettelbach/Germany: Psychometrica; 2018. Available from: <https://www.psychometrica.de/cNorm.html>
4. Lenhard W, Lenhard A, Gary S. Continuous norming (version 1.1.12) [software repository on the internet]; 2018. Available from: <https://github.com/WLenhard/cNORM>
5. Lumley, T. leaps: Regression subset selection (using Fortran code by Alan Miller, R package version 3.0) [Software]; 2017. Available from: https://cran.r-project.org/ web/packages/leaps/index.html.
6. Miller AJ. Subset selection in regression. Boca Raton: Capman & Hall/CRC; 2002.
7. Jenkins MA, Traub JF. Algorithm 419: zeros of a complex polynomial [C2]. Communications of the ACM. 1972Jan;15(2):97–9.
8. Brent RP. Algorithms for minimization without derivatives. Prentice-Hall series in automatic computation. Englewood Cliffs New Jersey; 1973
