# Supplementary figures and images for: Continuous norming of psychometric tests: A simulation study of parametric and semi-parametric approaches
Source: PLoS One. 2019 Sep 17;14(9):e0222279. doi: 10.1371/journal.pone.0222279 (PMC6748442; doi:10.1371/journal.pone.0222279)

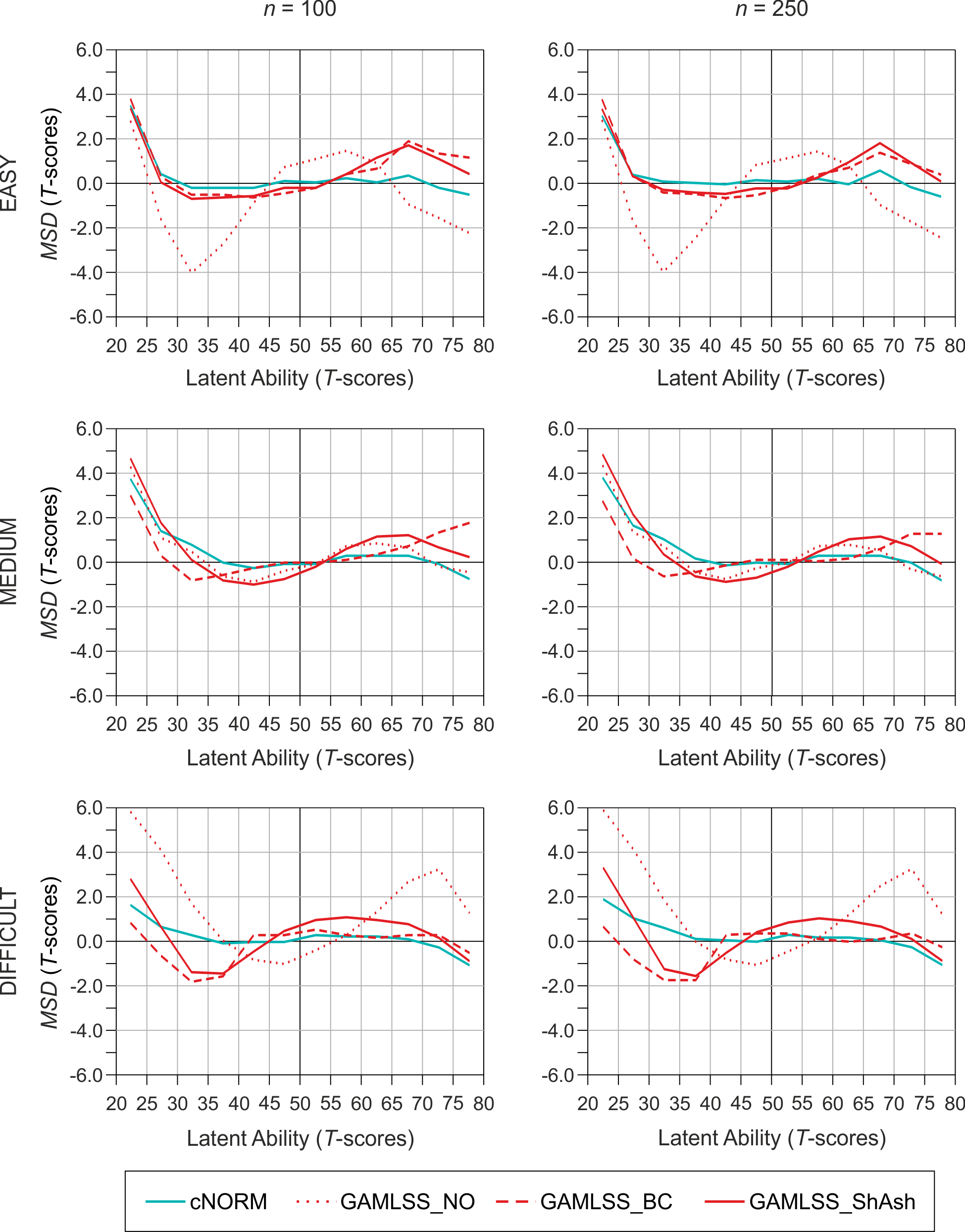

Supplement: S1 Fig — (TIF) [file pone.0222279.s006.tif]
